# Polyanion‐Type Electrode Materials for Sodium‐Ion Batteries

**DOI:** 10.1002/advs.201600275

**Published:** 2017-01-25

**Authors:** Qiao Ni, Ying Bai, Feng Wu, Chuan Wu

**Affiliations:** ^1^ Beijing Key Laboratory of Environmental Science and EngineeringSchool of Materials Science & EngineeringBeijing Institute of TechnologyBeijing100081P. R. China; ^2^ Collaborative Innovation Center of Electric Vehicles in BeijingBeijing100081P. R. China

**Keywords:** electrode materials, energy conversion, energy storage, polyanions, sodium‐ion batteries

## Abstract

Sodium‐ion batteries, representative members of the post‐lithium‐battery club, are very attractive and promising for large‐scale energy storage applications. The increasing technological improvements in sodium‐ion batteries (Na‐ion batteries) are being driven by the demand for Na‐based electrode materials that are resource‐abundant, cost‐effective, and long lasting. Polyanion‐type compounds are among the most promising electrode materials for Na‐ion batteries due to their stability, safety, and suitable operating voltages. The most representative polyanion‐type electrode materials are Na_3_V_2_(PO_4_)_3_ and NaTi_2_(PO_4_)_3_ for Na‐based cathode and anode materials, respectively. Both show superior electrochemical properties and attractive prospects in terms of their development and application in Na‐ion batteries. Carbonophosphate Na_3_MnCO_3_PO_4_ and amorphous FePO_4_ have also recently emerged and are contributing to further developing the research scope of polyanion‐type Na‐ion batteries. However, the typical low conductivity and relatively low capacity performance of such materials still restrict their development. This paper presents a brief review of the research progress of polyanion‐type electrode materials for Na‐ion batteries, summarizing recent accomplishments, highlighting emerging strategies, and discussing the remaining challenges of such systems.

## 1 Introduction

**Figure 1:**
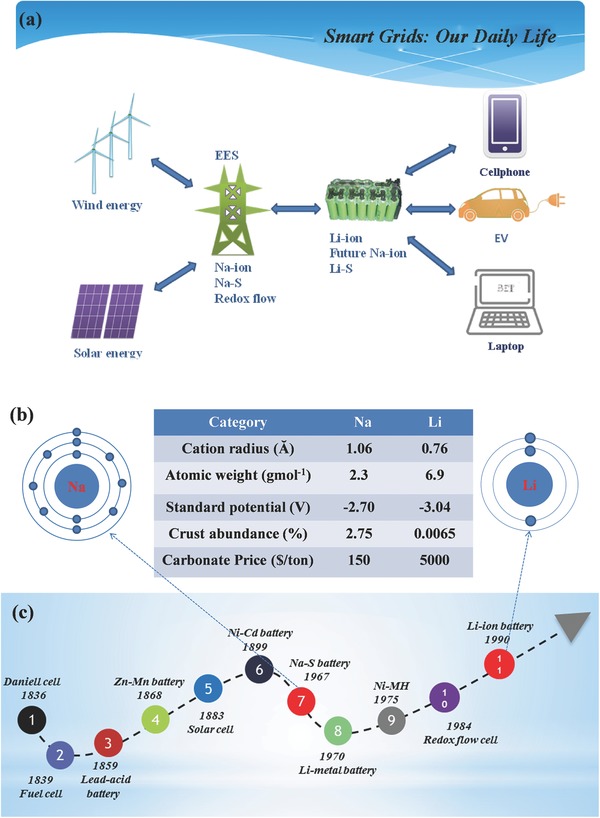
a) A simplified model for the relationship between renewable energy generation, grid, commercial secondary batteries and hybrid/electric vehicle transport. b) The comparison between Na and Li. c) The battery development history of the past 200 years.

So far, fossil fuels remain our primary power supply resource. However, extensive use of fossil fuels is the main cause of global warming because they emit large amounts of carbon dioxide. Therefore, the development and utilization of renewable energy such as solar and wind energy for power generation have become urgent. However, because normal operation of a power grid requires the stable and continuous generation of electricity and because both solar and wind energy are very dependent on environmental factors such as the weather, season, and location, they are currently considered unsuitable for modern grids. To overcome this problem, large‐scale electrochemical energy storage (EES) technologies based on batteries have been valued in recent years for their high round‐trip efficiency, flexible power, suitable energy characteristics to meet different grid functions, long cycle life, and low maintenance.^1^ **Figure** **1**a shows the stock‐flow diagram of renewable energy generation, EES, and energy sources needed for different electronic equipment and electric vehicle transports, all of which affect our daily lives.

According to data from the U.S. Geological Survey, the global lithium reserves in 2014 were approximately 13 million tons.^2^ The average annual demand for lithium carbonate (Li_2_CO_3_) will grow by 16.76% within the next six years; therefore, global lithium reserves without recycling can only last for 28 years. We can imagine that the demand will become astronomic if more electric vehicles are introduced because electric vehicles generally use a 60 KWh lithium‐ion battery pack. These data generate fear of a potential Li shortage and further price increases.^3^ Electrical energy storage technology is the key to the development of new energy sources and increased manufacture of electric vehicles. Nevertheless, batteries are closely related to the development of large‐scale renewable energy; thus, the resource‐depleting and price‐rising lithium resources cannot meet the requirements of increased industrial production.

Recently, much attention has been focused on room‐temperature Na‐ion batteries due to the cost‐effectiveness of sodium resources as a result of virtually limitless seawater. Although Na‐ion batteries have a similar charge–discharge principle as Li‐ion batteries, the larger cation radius and the heavier atomic weight combined with the higher standard potential of Na than that of Li generally result in an inferior reversible capacity and lower energy density (Figure 1b). However, the alkali metals of Na and Li lie in the same main group, and thus they have similar chemical performance, allowing much of the work that has been carried out for Li‐ion batteries to be equally applied to Na‐ion batteries. As depicted in Figure 1c, which summarizes the development of batteries over the past 200 years, studies of Na‐based batteries were carried out even earlier than those of Li‐based batteries. Nevertheless, the rapid expansion of Li‐ion batteries has resulted in minimal research on Na‐ion batteries.

Among the many anode and cathode materials available for Na‐ion batteries, such as layered oxides, polyanion‐type compounds, metal hexacyanometalates, and organic compounds, polyanion‐type compounds are perceived as one of the most promising for future Na‐ion batteries on account of their structural stability, safety, and appropriate operating potential. Taking phosphate as an example, it contains special tetrahedral PO_4_ units with strong covalent bonding, which results in the relative isolation of valence electrons from polyanions.^4^ This special three‐dimensional (3D) stereostructure is quite favorable to the intercalation and deintercalation behavior of Na ions because the smaller energy orbit leaps from the highest occupied molecular orbital (HOMO) to the lowest unoccupied molecular orbital (LUMO), frequently accompanied by multi‐electron mechanisms.^5^ Therefore, understanding the unique electronic structure is a good way to develop practical polyanion‐type electrical materials for Na‐ion batteries.^6^

Here we summarize the recent research progress and prospective future of polyanion‐type electrode materials for Na‐ion batteries. To have a good knowledge of such materials, special focus is given to the morphology and material modifications, together with the problems that remain to be solved. In addition, some strategies are summarized and proposed to enhance the electrochemical performance of polyanion‐type electrode materials. It is believed that this review will inform readers of the rationality and prominence of polyanion‐type electrode materials as powerful candidates for Na‐ion batteries.

## 2 Characteristics of the Structures and Properties of Polyanion‐Type Electrode Materials

Polyanion‐type electrode materials can be classified as a type of compounds that contain a series of tetrahedron anion units (XO_4_)*^n^* ^–^ or their derivatives (X*_m_*O_3_ *_m_*_+ 1_)*^n^* ^–^ (X = S, P, Si, As, Mo, or W) with strong covalent‐bonded MO*_x_* polyhedra (M represents a transition metal).^7^

In most of the polyanion‐type compounds, (XO_4_)*^n^* ^–^ not only allows fast ion conduction in an open framework that is selected for the working alkali ion on discharge, it can also stabilize the operative redox potentials of transition metals. Such a special framework consisting of two‐dimensional (2D) van der Waals bonding or 3D frameworks offer a significant advantage in terms of inserting/extracting alkali‐metal atoms.^8^ Compared to layered oxide compounds, the strong X—O bonding in polyanion‐type compounds can introduce ionicity in M—O bonding, and the weaker ionic bonding in M—O increases the distance between its antibonding orbitals vis‐à‐vis the Na/Na^+^ redox couple, leading to a higher redox potential. This is called the “inductive effect” in polyanion‐type electrode materials.^9^ Furthermore, the strong X—O covalent bonds greatly improve the stability of O in the lattice, thus increasing the safety of such materials, which make them more suitable for rechargeable secondary batteries.

Since the first report of an LiFePO_4_ cathode material by Padhi,^9^ olivine‐type‐structured or NASICON‐structured(Na Super Ionic Conductor structure) materials have been considered promising hosts for rechargeable secondary batteries, in which an MO_6_ (M = transition metal) octahedron shares corners or edges with an XO_4_ (X = S, P, Si, As, Mo, or W) tetrahedron. Such special framework compounds have been known to undergo topotactic insertion/extraction of mobile atoms,^8^ resulting in small volume changes during cycling and minimal structural rearrangement during alkali metal ion insertion/extraction in electrode materials. Thanks to their structural diversity and stability, combined with the strong inductive effect of polyanions, such electrode materials generally have suitable operating potential and outstanding cycling performance.

## 3 Recent Advances in Polyanion‐Type Electrode Materials for Na‐ion Batteries

### 3.1 Phosphates

As one of the most typical representatives of polyanion‐type compounds, phosphates have attracted significant attention. Olivine‐type‐structured NaMPO_4_ (Fe, Mn) and NASICON‐structured Na*_x_*M_2_(PO_4_)_3_ (M = V, Ti) represent the main phosphate compounds being researched for Na‐ion batteries due to their good electrochemical properties.

#### 3.1.1 NaMPO_4_ (M = Fe, Mn)

**Figure 2:**
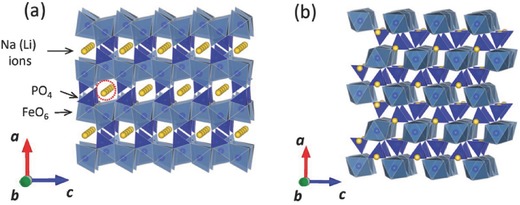
Crystal structure of phosphate‐based compounds with Fe: a) triphylite‐type Na(Li)FePO_4_; b) maricite‐type NaFePO_4_. Reproduced with permission.^10^ Copyright 2014, American Chemical Society.

NaFePO_4_, one of the earliest and most characterized polyanion‐type electrode materials for Na‐ion batteries, can be categorized into two different types of structures: triphylite‐type and maricite‐type (**Figure** **2**). Along the *b* direction, triphylite‐NaFePO_4_ has a one‐dimensional (1D) Na^+^ ion transport channel, whereas maricite‐NaFePO_4_ lacks transmission channels for the diffusion of sodium ions. Thus, maricite‐NaFePO_4_ has generally been considered an electrochemically inactive structure.

Owing to the lower diffusion coefficient of Na ions and the higher contact and charge‐transfer resistances in NaFePO_4_ cathodes, the rate performance of C‐NaFePO_4_ in Na‐ion batteries is much worse than that of C‐LiFePO_4_ in Li‐ion batteries. However, the cycling stability of C‐NaFePO_4_ is almost comparable to that of C‐LiFePO_4_, retaining 90% of its capacity even after 100 charge/discharge cycles at rate of 0.1 C.^11^

Conventional solid‐phase reactions at high temperatures are no longer suitable for synthesizing triphylite‐type NaFePO_4_ because the thermodynamically stable phase of NaFePO_4_ is not triphylite but maricite.^12^ Poul was the first to discover that the guest Li ions in the olivine iron phosphate host can be replaced by Na ions.^13^ Later, triphylite‐type NaFePO_4_ was frequently obtained via chemical or electrochemical displacement methods from triphylite‐type LiFePO_4_ in organic solutions.^11^, ^14^, ^15^

**Figure 3:**
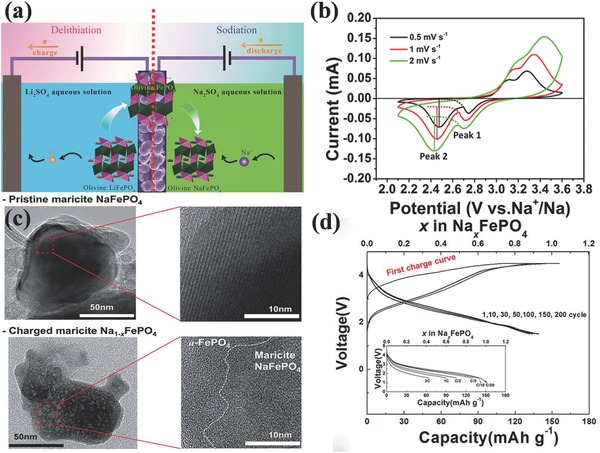
a) Synthetic scheme of the aqueous electrochemical displacement process from olivine LiFePO_4_ to isostructural NaFePO_4_. b) Cyclic voltammograms of NaFePO_4_/C electrode in 1 mol L^−1^ NaPF_6_/EC:DEC (1:1 in vol) solution at various scan rates. Reproduced with permission.^15^ Copyright 2015, American Chemical Society. c) Comparison of TEM images between pristine maricite NaFePO_4_ and partially charged maricite Na_1‐x_FePO_4_. This confirms the two‐phase reaction at transformation from maricite NaFePO_4_ to a‐FePO_4_ during the first charge. d) Galvanostatic curves of maricite NaFePO_4_ over 200 cycles at C/20 in a Na cell (inset: discharge curves of maricite NaFePO_4_ as a function of the C rate from C/20 to 3 C). Reproduced with permission.^16^ Copyright 2014, Royal Society of Chemistry.

Cao prepared a triphylite‐type NaFePO_4_/C microsphere cathode by a two‐step aqueous electrochemical transition process from an LiFePO_4_/C precursor,^15^ LiFePO_4_ as the working electrode, activated carbon as the counter electrode, and Ag/AgCl as reference electrode (**Figure** **3**a). The obtained NaFePO_4_/C cathode showed a high discharge capacity of 111 mAh g^−1^ and excellent cycling stability, with 90% capacity retention over 240 cycles at 0.1 C. Moreover, the existence of a Na_2/3_FePO_4_ intermediate was first observed during the Na^+^ intercalation process with conventional electrochemical techniques. Figure 3b shows the cyclic voltammetry profile. At scan rates from 0.5 mV s^−1^ to a high rate of 2 mV s^−1^, the two well‐defined reductive peaks indicate an identical two‐step phase transition reaction. Recently, maricite‐type NaFePO_4_ was reinvestigated, and for the first time, the Na extraction/insertion was proven to be reversible in the maricite NaFePO_4_ electrode, in contrast to the conventional view that maricite NaFePO_4_ is electrochemically inactive. Quantum mechanics calculations (the PBE functional of density functional theory) and experiments were combined to identify the electrochemical mechanism responsible for the electrochemical activity of maricite NaFePO_4_.^16^ The investigation on the Na^+^ re‐(de)intercalation mechanism revealed that all Na ions were deintercalated from the nano‐sized maricite NaFePO_4_. X‐ray diffraction (XRD) and extended X‐ray absorption fine structure (EXAFS) analyses showed that after the first deintercalation of Na ions, maricite FePO_4_ transformed into a‐FePO_4_ (Figure 3c), which allowed substantially smaller barriers for Na to hop from site to site. Amorphous FePO_4_ formed after maricite NaFePO_4_ fully desodiated, which delivered a capacity of 142 mAh g^−1^ (92% of the theoretical value) at the first cycle, and it showed outstanding cyclability, with a 95% capacity retention of the initial cycle after 200 cycles (Figure 3d).

Similar to NaFePO_4_, NaMnPO_4_ also has two structure modifications: maricite‐type and olivine‐type structures. Both structures consist of layers composed of corner‐sharing metal octahedra bridged through the oxygen atoms in PO_4_^3−^ groups. The hydrothermal method was reported to have been used for the preparation of maricite NaMnPO_4_ single crystals at 420 °C for 6 days, and a solid‐state reaction was reported for the synthesis of olivine NaMnPO_4_ at 400 °C for 6 h then at 900 °C for 24 h.^17^ However, neither of these two structural modifications have shown favorable electrochemical performance, even though new synthesis methods such as thermal decomposition and ion exchange have been reported.^18^ Recently, based on an ion‐exchange reaction, olivine‐type NaMnPO_4_ manifested a reversible capacity of 80–85 mAh g^−1^, corresponding to 0.5 Na intercalation. Although the electrochemical performance of NaMnPO_4_ seems to be unsatisfactory, it is likely to exhibit more ion intercalation by optimizing the structure and external electrolyte solutions.^19^

#### 3.1.2 NASICON‐Structured Na_x_M_2_(PO_4_)_3_ (M = V, Ti; x = 1,2,3)

NASICON‐structured materials were first reported as solid‐state electrolytes by Yao.^20^ NASICON‐structured Na*_x_*M_2_(PO_4_)_3_ (M = V, Ti; *x* = 1,2,3) is a kind of fast ion conductor with open 3D ion transport channels and high ion diffusion rates. As variable valence metal ions in the NASICON structure, this special framework was first reported by Goodenough and his co‐workers, who researched the potential variation of V^4+^/V^3+^, V^3+^/V^2+^, Fe^3+^/Fe^2+^, Nb^5+^/Nb^4+^, and Nb^4+^/Nb^3+^ in the process of lithium insertion/extraction.^8^, ^21^

#### 3.1.3 NASICON‐Structured Na_3_V_2_(PO_4_)_3_

**Figure 4:**
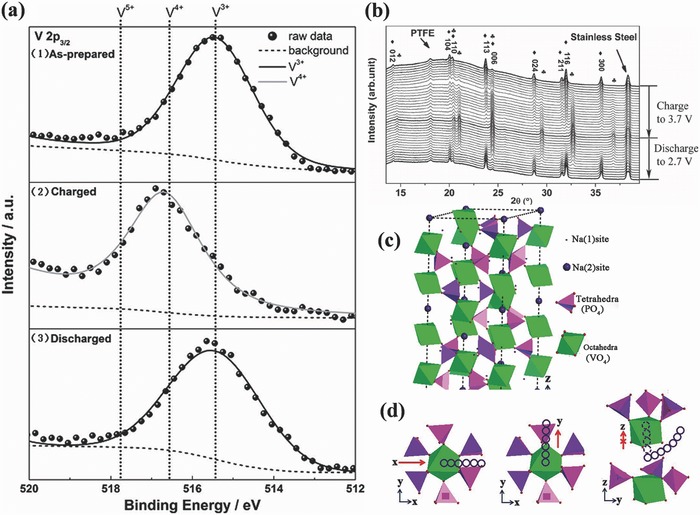
a) Ex‐situ XPS studies of NVP/C electrodes: 1) pristine sample, 2) charged, 3) discharged. Reproduced with permission.^25^ b)In‐situ XRD patterns of the Na_3_V_2_(PO_4_)_3_/Na cell cycled between 3.7 and 2.7 V at a current rate of C/10, ♦ Na_3_V_2_ (PO_4_)_3_, ♣ NaV_2_ (PO_4_)_3_. Reproduced with permission.^26^ c) Schematic representation of the Na_3_V_2_(PO_4_)_3_ structure. d) Possible Na ion migration paths in Na_3_V_2_ (PO_4_)_3_ along *x*, *y* and curved *z* directions. Reproduced with permission.^27^ Copyright 2014, Royal Society of Chemistry.

Na_3_V_2_(PO_4_)_3_ as a fast Na^+^‐transportable NASICON framework has attracted much attention as a promising cathode material for Na‐ion batteries.^22^, ^23^, ^24^ Chen and his group first reported the fabrication of carbon‐coated Na_3_V_2_(PO_4_)_3_ as a novel electrode material for Na‐ion batteries by a one‐step solid‐state reaction.^22^ It showed a flat voltage plateau at 3.4 V vs. Na^+^/Na in a non‐aqueous Na‐ion battery. Its initial charge and discharge capacities were 98.6 and 93 mAh g^−1^, respectively, which demonstrated that carbon coating can significantly improve the sodium storage performance. In order to determine the mechanism of sodium insertion/extraction into/out of the Na_3_V_2_(PO_4_)_3_ lattice, both ex‐situ X‐ray photoelectron spectroscopy (XPS) (**Figure** **4**a) and in‐situ XRD (Figure 4b) were carried out.^25^, ^26^ The results indicated that the mechanism of sodium insertion/extraction can be ascribed to a kind of typical two‐phase reaction at 3.4 V. The results also showed that all peaks from Na_3_V_2_(PO_4_)_3_ were maintained and that the intensities of their peaks gradually decreased, indicating a typical two‐phase reaction between Na_3_V_2_(PO_4_)_3_ and NaV_2_(PO_4_)_3_. In an effort to understand the 3D characteristics of the internal ion transportation paths of Na_3_V_2_(PO_4_)_3_, first‐principles calculations combined with experiments were conducted by evaluating the activation energies towards Na_3_V_2_(PO_4_)_3_. It was proven that two pathways along the *x* and *y* directions and one possible curved route for ion migration were favored with 3D transport characteristics (Figure 4c and d), providing ample evidence for the theoretical capacity of 117 mAh g^−1^.^27^

Despite many advantages associated with Na_3_V_2_(PO_4_)_3_, such as high stability and relatively high voltage, low conductivity is still the key drawback to its commercial application. In the past few years, researchers have investigated many different ways to overcome this problem, mainly concentrating on optimizing the synthetic strategies,^23^, ^28^, ^29^ surface‐conducting modifications,^22^, ^30^, ^31^, ^32^ element doping, and so forth.^33^, ^34^, ^35^, ^36^

To optimize the morphology and further improve the electronic conductivity and structural stability of Na_3_V_2_(PO_4_)_3_, various synthetic strategies such as traditional solid‐state reactions, sol–gel processing, the electrospinning method, and hydrothermal and solvothermal processing routes have all been attempted. Recently, a solvothermal processing method named “facile self‐sacrificed route” for synthesizing a 3D NVP nanofiber framework was reported.^23^ For the first time, an outside–in morphological evolution mechanism was proposed based on time‐dependent experiments. The controllably constructed NVP cathode material showed outstanding cycling stability and rate performance in both a sodium half‐cell and a full battery. Through electrospinning, a 1D nanostructured Na_3_V_2_(PO_4_)_3_ material was synthesized.^29^ The Na_3_V_2_(PO_4_)_3_ nanoparticles were uniformly encapsulated in 1D carbon nanofibers, which greatly shortened the ion diffusion path and increased the elelctrode/electrolyte contact area.

**Figure 5:**
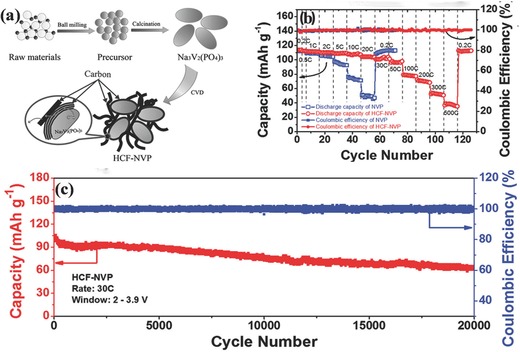
a) Schematic illustration of the synthesis of hierarchically carbon‐coated Na_3_V_2_(PO_4_)_3_ (HCF‐NVP). b) Rate capability of the NVP and HCF‐NVP electrodes. c) Long‐term cycling performance of the HCF‐NVP electrode at a high current rate of 30 C after 20 000 cycles: voltage window is 2–3.9 V. Reproduced with permission.^32^

Surface modification has been widely used to improve the electronic conductivity of Na_3_V_2_(PO_4_)_3_. Carbon coatings are particularly attractive because of their high conductivity, even using carbon concentrations as low as 0.5–10 wt%.^32^ Furthermore, their low cost, simplicity of introduction during or after the synthesis of Na_3_V_2_(PO_4_)_3_, and chemical stability all promote the wide application of such coatings in surface modification. Various carbon sources and different carbon frameworks have been proposed to form the carbon coating layer. Since the excellent cycling stability and superior rate capability of Na_3_V_2_(PO_4_)_3_ was first reported for Na‐ion batteries using cationic surfactants as carbon resource,^25^ different kinds of carbon coating methods have emerged. In an attempt to achieve both high rate capability and stable cyclability, another effective strategy for surface modification is to embed Na_3_V_2_(PO_4_)_3_ particles in highly conductive and interconnected carbon frameworks. However, coated carbon formed from pyrolysis of an organic precursor is usually in an amorphous state with low electric conductivity. Recently, hierarchical carbon framework‐wrapped Na_3_V_2_(PO_4_)_3_ was synthesized using simple and cost‐effective chemical vapor deposition (CVD) (**Figure** **5**);^32^ CVD provides a facile and convenient method for generating highly conductive carbon with tunable dimensions such as 1D graphene sheets and 2D nanotubes.^37^ The hierarchical carbon framework consisted of graphene‐like coating layers and interconnected nanofibers, a structure that demonstrated a close‐to‐theory reversible capacity at 0.2 C, a superior high‐rate capability of 38 mAh g^−1^ at 500 C, and 54% capacity retention after 20 000 cycles at 30 C. Interestingly, only a negligible amount (0.73%) of carbon coating was contained in the NVP products.

**Figure 6:**
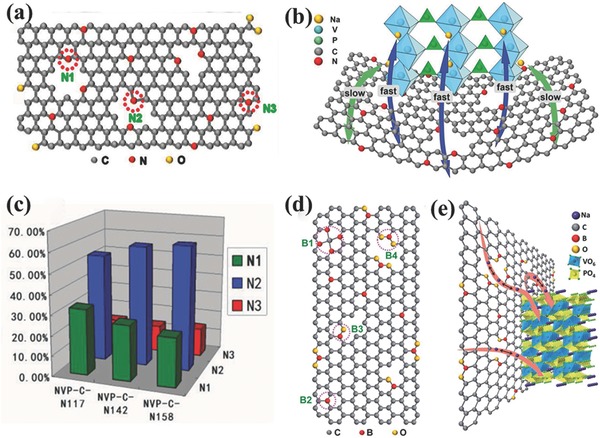
a) Schematic structure of the binding conditions of N in a carbon lattice. b) A schematic illustration of sodium ion storage mechanism in the NVP‐C‐N composite electrode. c) The histogram for ratio of different N species in various NVP‐C‐N samples. Reproduced with permission.^39^ d) Schematic structure of the bonding conditions of B in a carbon lattice, B1: B4C, B2: BC3, B3: BC2O and B4: BCO2. e) Schematic illustration of sodium ion storage mechanism in the NVP–C–B composite electrode. Reproduced with permission.^40^ Copyright 2015, Royal Society of Chemistry.

Element doping is considered to be another way to optimize the electrochemical performance of Na_3_V_2_(PO_4_)_3_, and it has been proven to be effective in improving the intrinsic electronic conductivity in Li‐ion batteries.^38^ In a recent study, Mg was selected to dope the V sites of a Na_3_V_2_(PO_4_)_3_ cathode material because of its light atomic weight and it potential for improving the performance of the electrode materials in Na‐ion batteries.^35^ As compared to undoped Na_3_V_2_(PO_4_)_3_, Mg‐doped Na_3_V_2−_ *_x_*Mg*_x_*(PO_4_)_3_/C composites prepared through a simple sol–gel method exhibited obvious enhancement of the electrochemical performance in terms of both the rate capability and the cycle performance. In order to improve the efficiency of carbon coatings and to further to enhance the conductivity of the electrode material layer on the surface of carbon, Shen et al. introduced the nonmetallic element nitrogen into the carbon layer.^39^ The nitrogen‐doped, carbon‐coated Na_3_V_2_(PO_4_)_3_ cathode material exhibited a remarkable improvement in Na storage properties, especially its rate performance. Furthermore, the modification effects of various nitrogen types on the electrochemical performance were explored in detail. The results demonstrated that nitrogen doping can introduce the necessary defects into the carbon coating layer to facilitate Na storage in the electrode materials (**Figure** **6**a and b). As depicted in Figure 6c, the optimized sample NVP‐CN142 had the greatest total mount of N1+N2, resulting in the greatest number of extrinsic defects introduced into the carbon lattice and thus the greatest possibility for accelerating Na^+^ diffusion. In the same way, the nonmetallic element B has also been used to dope carbon coating layers. Four different B‐doping species (B1, B2, B3, B4) are normally obtained in the carbon layer (Figure 6d and e).^40^ Thus, appropriately B‐ and N‐doped samples can create large numbers of extrinsic defects and active sites, which are beneficial to the fast diffusion of Na^+^ in the carbon layers. Familiar nonmetallic elements such as S, P, and Cl may have similar effects on the carbon layers.

#### 3.1.4 NASICON‐Structured NaTi_2_(PO_4_)_3_

**Figure 7:**
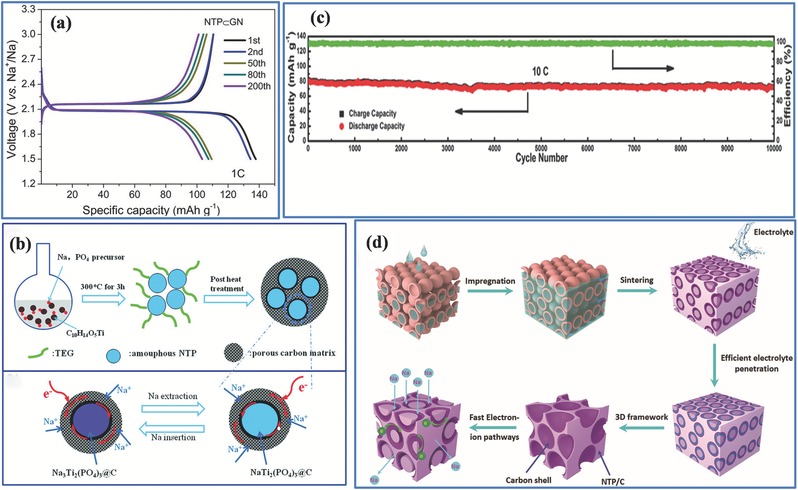
a) The discharge–charge profiles of the NTP ⊂ graphene network particle electrodes at 1C. Reproduced with permission.^49^ Copyright 2015, American Chemical Society. b) Schematic illustration of the synthesis of the NTP@C@PC nanocomposites and the reversibility of electrochemical reactions. Reproduced with permission.^50^ Copyright 2015, Royal Society of Chemistry. c) Ultra‐longterm cycling performance of rutile TiO_2_ highly regular NaTi_2_(PO_4_)_3_ (C/NTP‐RT) nanocubes electrode at a high current density of 10 C. Reproduced with permission.^46^ d) Schematic fabrication process for the frogspawn‐inspired NaTi_2_(PO_4_)_3_–C array. Reproduced with permission.^48^ Copyright 2015, Royal Society of Chemistry.

As a typical NASICON‐structured material, the titanium‐based NASICON compound NaTi_2_(PO_4_)_3_ has also been extensively researched as a Na‐ion battery anode material since it was first reported by Delmas.^41^ The TiO_6_ octahedra structures are connected by PO_4_ tetrahedra, which constitute the NASICON framework with two sodium ions. This unique NASICON‐type NaTi_2_(PO_4_)_3_ exhibited a higher theoretical capacity of 133 mAh g^−1^, with a typical two‐phase reaction between NaTi_2_(PO_4_)_3_ and Na_3_Ti_2_(PO_4_)_3_. As shown in **Figure** **7**a, a well‐defined redox plateau (≈2.1 V vs. Na^+^/Na) is fixed by the redox potential of Ti^4+^/Ti^3+^, which is high enough to avoid the formation of a surface electrolyte interphase (SEI).

As compared to other Na‐ion battery anode materials such as hard carbon,^42^ metal oxide,^43^ and intermetallic anode materials,^44^ NaTi_2_(PO_4_)_3_ (NTP) in an aqueous system combines the advantages of fast ion transport, slight volume expansion, low cost, high safety, and environmental friendliness, all of which contribute its commercialization. However, similar to other NASICON‐type materials, the practical applications of NTP are severely hindered by its poor rate performance owing to its low electronic conductivity.

In an effort to develop stability, Yu et al. fabricated NASICON‐type NTP with a high rate capability and long cycle life by preparing a “double carbon coating” through a soft‐chemical method.^45^ The “double carbon coating” design possesses the combined advantages of a suitable carbon coating layer thickness and smaller electroactive material particles, both contributing to fast Na^+^/e^−^ transfer in the nanocomposites and negligible structural variation during charge and discharge (Figure 7b). NTP/C with such a porous 3D carbon matrix delivered a reversible capability of 103 mAh g^−1^ at 5 C after 5000 cycles, and a 64 mAh g^−1^ capacity retention even at a high rate at 50 C. In an attempt to control the morphology of NTP, highly regular NTP nanocubes with a synergistic nanocoating of rutile TiO_2_ and carbon were prepared for Na‐ion batteries.^46^ This C/NTP‐RT electrode exhibited a high rate and an ultralong life: a superior capacity of 83.5 mAh g^−1^ at a rate of 10 C, and a cyclic capacity retention of 89.3% after 10 000 cycles (Figure 7c). Even more interesting is that Nature provides a variety of intricate 3D structures that can be duplicated in the laboratory.^47^ Inspired by the special “core–shell” structure of frog and fish spawn, a novel core–shell framework for NTP was designed via a facile impregnation process. A hollow carbon sphere was first built, followed by filling hollow carbon spheres with NTP nanospheres. As depicted in Figure 7d, the compact array had hierarchical pores and ordered channels. Electrochemical tests demonstrated that the NTP anions exhibited a favorable rate performance and superior cycle stability, ascribed to the fast electron transport and superior Na^+^ intercalation in the 3D “core–shell” structure.^48^ NTP has attracted broad attention due to its stability, abundance, low cost, and environmentally benign characteristics. If other transition metal elements such as Fe are substituted for some part of Ti, different insertion mechanisms may take place, which deserve further investigation.

### 3.2 Pyrophosphates

Since Li_2_FeP_2_O_7_ was first introduced as a cathode material for Li‐ion batteries, different kinds of pyrophosphates have shown substantially improved kinetics as compared to other phosphate‐based materials, including LiFePO_4_.^51^, ^52^ Nevertheless, as the most promising large‐scale storage system, Na‐ion batteries have been researched extensively over the last few years, along with Na‐based pyrophosphates such as NaMP_2_O_7_ (M = Ti, V, Fe),^53^, ^54^, ^55^, ^56^, ^57^ Na_2_MP_2_O_7_ (M = Fe, Mn, Co),^58^, ^59^, ^60^ and Na_4_M_3_(PO_4_)_2_P_2_O_7_ (M = Fe, Co, Mn).^61^ Na_2_MP_2_O_7_ (M = Fe, Mn, Co) has different structural configurations, i.e., triclinic (space group *P*1), monoclinic (space group *P*2_1_/*C*), and tetragonal (space group *P*4_2_/*mnm*). These three different structures of Na_2_MP_2_O_7_ (M = Fe, Mn, Co) all contribute to the Na^+^ transport direction channel.

#### 3.2.1 NaMP_2_O_7_ (M = Fe, Ti, V)

**Figure 8:**
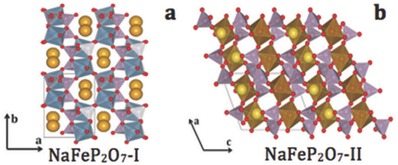
The rich crystal chemistry observed in sodium metal pyrophosphates. Monoclinic structured polymorphs of a) I‐NaFeP_2_O_7_ and b) II‐NaFeP_2_O_7_. (a,b) Reproduced with permission.^52^

NaFeP_2_O_7_ consists of two different structures due to irreversible phase transitions at different temperatures. It has been pointed out that low‐temperature I‐NaFeP_2_O_7_ is isostructural with KAlP_2_O_7_, which was reported by Calvo.^62^ I‐NaFeP_2_O_7_ has been found to be thermally unstable: when the temperature increases to 750 °C, it forms II‐NaFeP_2_O_7_ (**Figure** **8**). Crystalline II‐NaFeP_2_O_7_ was prepared by a high‐temperature solid‐state melting method.^53^ The precursor mixture was first heated to 900 °C and then melted at 1100 °C for 1 h. The prepared crystals of II‐NaFeP_2_O_7_ were then obtained after quenching the melted mixture. It was reported that the corner‐sharing P_2_O_4_ and FeO_3_ octahedral units constituted the main skeleton of the FeP_2_O_7_ structure. The PO_4_ tetrahedron shared its corners with the other three octahedra and one tetrahedron, whereas the FeO_6_ octahedron shared all its apices with the PO_4_ tetrahedra (Figure 8b). Furthermore, such a special structure, comprising FeO_3_ octahedral layers and P_2_O_4_ tetrahedral layers, allows Na^+^ ions to migrate in the plane parallel to (001).

Similar compounds such as NaTiP_2_O_7_ and NaVP_2_O_7_ have also been reported.^55^, ^56^, ^57^, ^63^ For example, titanium(III) pyrophosphate NaTiP_2_O_7_ has two forms, α‐NaTiP_2_O_7_ and β‐NaTiP_2_O_7_. The special intersecting tunnel structure is considered to be available for the sodium ion (de)insertion. The feasibility of NaVP_2_O_7_ as a high‐voltage cathode material for Na‐ion batteries (3.4 V) fabricated via a conventional solid‐state reaction was first reported by Kee.^56^ It showed an initial discharge capacity of 38.4 mAh g^−1^ (the theoretical capacity = 108 mAh g^−1^) at 0.05 C within the potential window of 2.5–4.0 V (vs. Na^+^/Na). The limited electrochemical activity of NaVP_2_O_7_ is thought to account for its intrinsically high resistance, which restricts phase‐transition kinetics between NaVP_2_O_7_ and Na_1−_ *_x_*VP_2_O_7_.

#### 3.2.2 Na_2_MP_2_O_7_ (M = Fe, Co, Mn, Cu)

**Figure 9:**
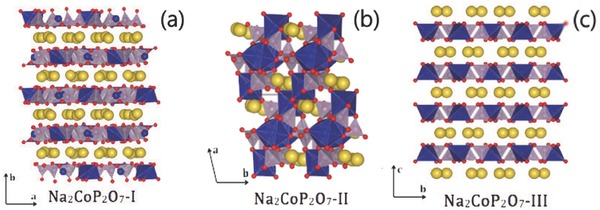
Polymorphism in Na_2_CoP_2_O_7_: a) orthorhombic (*P*21/*cn*), b) triclinic (*P*‐1) and c) tetragonal (*P*42/mnm) forms. Co interestingly forms CoO_6_ octahedra or CoO_4_ tetrahedra. Reproduced with permission.^52^

The family of Na_2_MP_2_O_7_ (M = Fe, Co, Mn, Cu) can be classified according to structure: triclinic, tetragonal, and monoclinic. The different crystal structures of Na_2_CoP_2_O_7_ are shown in **Figure** **9**. Furthermore, **Table** **1** shows the polymorphism structures with cell parameters and space groups of Na_2_MP_2_O_7_ (M = Fe, Co, Mn, Cu) reported so far.

**Table 1:** Unit cell parameters of polymers for Na_2_MP_2_O_7_ (M = Fe, Co, Mn, Cu)

	Structure	a/Ă	b/Ă	c/Ă	β/°	Vol/Ă^3^	Ref.
Na_2_FeP_2_O_7_	triclinic	6.4299	9.4145	11.0110	85.465	573.39	[[qv: 58a]]
Na_2_CoP_2_O_7_	triclinic	9.735	10.940	12.289	121.76	566.8	59, 65
	orthorhombic	15.4061	10.2885	7.7031	–	1221.0	[[qv: 64a]]
	tetragonal	7.706	10.301	–	–	–	[[qv: 64b]]
Na_2_MnP_2_O_7_	triclinic	5.316	6.580	9.409	95.25	290.96	[[qv: 58c,66]]
β‐Na_2_MnP_2_O_7_	triclinic	9.922	11.086	12.473	121.94	599.4	[[qv: 58b]]
α‐Na_2_CuP_2_O_7_	monoclinic	8.823	13.494	5.108	92.77	607.5	[[qv: 64c]]
β‐Na_2_CuP_2_O_7_	monoclinic	14.728	5.698	8.067	115.15	612.8	[[qv: 64c]]

Among these compounds, sodium iron pyrophosphate (Na_2_FeP_2_O_7_), the first compound ever reported in the pyrophosphate family for Na‐ion batteries, maintained a well‐defined channel structure and exhibited a reversible capacity of 90 mAh g^−1^ with good cycling performance.^67^ Pure Na_2_FeP_2_O_7_/C powders were obtained through a high‐temperature solid‐state method. The as‐synthesized powder was then carbon coated through a dry ball‐milling process. The chemical reaction is as follows:

(1) $$\begin{array}{l} {\mathrm{Na}}_{2}{\mathrm{CO}}_{3}\;+\;2{\left({\mathrm{NH}}_{4}\right)}_{2}{\mathrm{HPO}}_{4}\;+\;{\mathrm{FeC}}_{2}O_{4}\cdot 2H_{2}O\\ \;\;\;\;\;\;\to {\mathrm{Na}}_{2}{\mathrm{FeP}}_{2}O_{7}\;+\;2N_{2}\;+\;3\mathrm{CO}\;+\;7H_{2}O \end{array}$$

**Figure 10:**
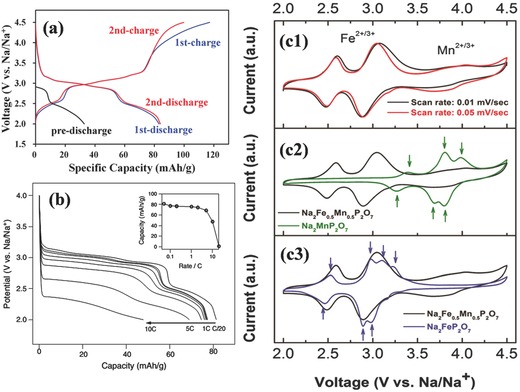
a) The voltage profiles starting with the discharge (pre‐discharge). Reproduced with permission.^67^ b) The discharge capacity of Na_2_FeP_2_O_7_ as a function of rate is plotted to show the kinetics (Inset: the capacity as a function of discharge rate is given). Reproduced with permission.^70^ Copyright 2016, Elsevier. c) Cyclic voltammetry (CV) data for Na_2_Fe_0.5_Mn_0.5_P_2_O_7_, Na_2_MnP_2_O_7_ and Na_2_FeP_2_O_7_. Reproduced with permission.^71^ Copyright 2016, Royal Society of Chemistry.

Synchrotron X‐ray diffraction (SXRD) patterns combined with DFT calculation were used to study the crystal structure of Na_2_FeP_2_O_7_, and the results indicated that eight sodium sites were contained in the unit cells.^67^ According to DFT calculations, the energy of the Na1 site was higher than those of the Na2–Na8 sites, and the migration barrier required for the extraction of Na1 (0.48 eV) along the [011] channel direction was also lower than those of other extraction channels (0.54–0.67 eV). Therefore, Na1 is the most easily accessible Na site for (de)intercalation, both thermodynamically and kinetically. The charge/discharge profile in **Figure** **10**a shows a staircase‐type voltage profile with two plateaus at 2.5 V and 3 V. The charging capacity of 130 mAh g^−1^ (higher than the theoretical capacity of 97 mAh g^−1^, corresponding to the activity of almost one Na ion per each Na_2_FeP_2_O_7_) was observed. However, because of the higher redox potential (≈5 V) of Fe^3+^/Fe^4+^ over the potential window of 2.0–4.5 V, the possibility of the extraction of more than one Na ion should be excluded. Therefore, some unwanted side reactions may result from this. Even with increasing the rate from C/20 to 10 C, the two characteristic plateaus at 2.5 V and 3.0 V remained, and the Na_2_FeP_2_O_7_ cathode exhibited excellent rate kinetics, with 91% and 85% of the initial capacity retained at 1 C and 5 C, respectively. Further studies are expected to focus on developing the electrochemical performance of Na_2_FeP_2_O_7_ using methods such as controlling the particle size, surface coating techniques, or doping with other metal elements.

Na_2_MnP_2_O_7_ was first reported by Huang in 1998.^66^ In general, similar to the case with polyanions, Na‐ion battery cathodes are considered to have poorer activity than their Li‐ion battery counterparts. However, Na_2_MnP_2_O_7_ has been proven to be far superior as compared to its almost inactive Li counterpart (Li_2_MnP_2_O_7_). Unlike most Mn‐based cathode materials, which suffer severely from poor kinetics, Na_2_MnP_2_O_7_ has shown good electrochemical performance, even at a high potential of 3.8 V (vs. Na/Na^+^).^68^ A reversible capacity of 90 mAh g^−1^ was obtained in the voltage range of 1.5−4.5 V (vs. Na/Na^+^) at a rate of C/20 (based on the theoretical capacity of 97.5 mAh g^−1^), with 96% capacity retention after 30 cycles and 70% capacity retention at a rate increase from 0.05 C to 1 C. On the basis of first‐principles calculations, it was proven that the small scale of atomic rearrangements in Na_2_MnP_2_O_7_ can lower the barriers for electron conduction and phase boundary migration, which causes multiple bonds to be broken and reformed during charging reactions in Li_2_MnP_2_O_7_.^60^, ^68^ However, Na_2_MnP_2_O_7_ has been shown to have an inferior rate capability due to the intrinsic lower electronic conductivity of Mn.^69^ Therefore, a mixed transition metal pyrophosphate material, Na_2_Fe_0.5_Mn_0.5_P_2_O_7_, was synthesized by substituting some Mn with Fe.^71^ Ex situ XRD and cyclic voltammetry (CV) analyses indicated that Na_2_Fe_0.5_Mn_0.5_P_2_O_7_ is a single‐phase reaction due to different Na site occupancy as compared to the biphasic reaction of Na_2_FeP_2_O_7_ and Na_2_MnP_2_O_7_. As depicted in Figure 10c, Na_2_FeP_2_O_7_ and Na_2_MnP_2_O_7_ have two‐phase transitions at ≈3.8 V and ≈3 V, respectively.^68^ Such mixed iron–manganese sodium‐based pyrophosphate cathodes may pave the way to expanding the research scope of pyrophosphates for Na‐ion batteries.

The diphosphate Na_2_CoP_2_O_7_ was first reported by Erragh and his group through the solid‐state method.^65^ Further research demonstrated that it exists in three different crystal structures: triclinic (*P*1), orthorhombic (*P*2_1_ *cn*), and tetragonal (*P*4_2_/*mnm*), shown in Figure 9. Layer‐structured orthorhombic Na_2_CoP_2_O_7_ with two‐dimensional channels for the migration of Na^+^ ions has also been reported to be a suitable cathode material for Na‐ion batteries.^72^ After cycling at a rate of C/20, a reversible capacity of 80 mAh g^−1^ (theoretical capacity of 96.11 mAh g^−1^) and an average operating potential of 3.0 V (vs. Na/Na^+^) were obtained. Surprisingly, no distinct Co^3+^/Co^2+^ redox plateau emerged; instead, it exhibited continuously sloping voltage profiles. This may be ascribed to reversible multistep phase transitions and structural ordering during sodium (de)insertion and the involvement of large hybridization with a wide band of oxygen. However, the cycle performance is unsatisfactory due to the high operating potential (4.5 V), which leads to the decomposition of the electrolyte and the accumulation of organic components at the surface of the Na_2_CoP_2_O_7_.

#### 3.2.3 Na_4_M_3_(PO_4_)_2_P_2_O_7_ (M = Fe, Co, Ni)

The mixed phosphates Na_4_M_3_(PO_4_)_2_P_2_O_7_ (M = Fe, Co, Ni) are also promising class of cathode materials for Na‐ion batteries as they exert small volume changes and exhibit good cycle performance. So far, the Na_4_Co_3_(PO_4_)_2_P_2_O_7_ system has been prepared by a typical sol–gel method at a high potential window of 4.1–4.7 V (vs. Na^+^/Na).^73^ It delivered a capacity of 95 mAh g^−1^ at a rate of 0.2 C; even at a high rate of 25 C, it exhibited small polarization in the charge–discharge process.

The most representative mixed phosphate is Na_4_Fe_3_(PO_4_)_2_P_2_O_7_. XRD patterns show that the structure of the as‐prepared Na_4_Fe_3_(PO_4_)_2_P_2_O_7_ can be indexed as orthorhombic (space group *Pn*2_1_ *a*).^74^ The lattice parameters are *a* = 18.03744(11) Å, *b* = 6.52727(4) Å, *c* = 10.64413(7) Å, and V = 1253.189(1) Å^3^. It delivers approximately 82% of the theoretical capacity (129 mAh g^−1^) at approximately 3.2 V (vs. Na/Na^+^). In order to further understand the electrochemical reaction mechanism during charge and discharge, first‐principles calculations were used to investigate the intermediate states of Na*_x_*Fe_3_(PO_4_)_2_P_2_O_7_ (1 < *x* < 4). The results indicated that Na_4_Fe_3_(PO_4_)_2_P_2_O_7_ does not undergo a phase change upon Na ion extraction, and thus it undergoes a small volume change of less than 4%.^74^ In an effort to further probe the defect, diffusion, and voltage trends of the Na_4_M_3_(PO_4_)_2_P_2_O_7_ (M = Fe, Co, Ni) class of materials, a combination of atomistic energy minimization, molecular dynamics (MD), and DFT simulation techniques were carried out.^75^ First, atomistic energy minimization demonstrated that the Fe‐based material had the highest defect concentration, which we know has a significant impact on electrochemical performance;^39^ second, MD simulations suggested that the Na^+^ diffusion coefficients and activation barriers compared favorably to those of a Li‐ion battery cathode; and third, the DFT simulation techniques demonstrated that doping Ni in Na_4_Fe_3_(PO_4_)_2_P_2_O_7_ increased the operating potential significantly.

### 3.3 Fluorine Transition Metal Salts: NaM(XO_4_)F (M = Fe, Co, V, Mn; X = P, S)

To improve the electrochemical performance of Na‐ion batteries, different methods have been attempted, such as increasing the specific capacity by preparing nanocomposites with carbon‐related materials,^76^ and designing aporous networks.^77^ Increasing the working potential of cathode materials may be another effective strategy to improving the energy density of Na‐ion batteries. Thanks to the inductive effect of the PO_4_^3−^ and SO_4_^3−^ groups and the high electronegativity of the F^−^ anion, both fluorophosphates and fluorosulfates are considered to be promising high‐potential cathode materials.

#### 3.3.1 Fluorophosphates

**Figure 11:**
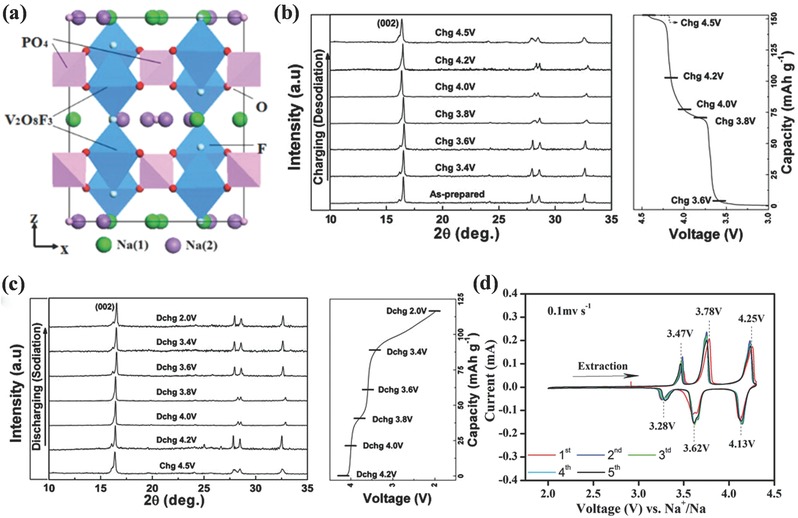
a) Schematic representation of the Na_3_V_2_(PO_4_)_2_F_3_ structure viewed along the y axis. Reproduced with permission.^84^ Copyright 2014, American Chemical Society. b,c) Ex situ analysis of Na_3_V_2_(PO_4_)_2_F_3_ electrodes during charge (b) and discharge (c). Reproduced with permission.^91^ Copyright 2012, Royal Society of Chemistry. d) Cyclic voltammetry of Na_3_V_2_(PO_4_)_2_F_3_ at a scan rate of 0.1 mV s^–1^ (vs. Na^+^/Na). Reproduced with permission.^85^ Copyright 2015, Royal Society of Chemistry.

Recently, a variety of fluorophosphates have been reported as Na‐ion battery cathode materials, such as Na_2_FePO_4_F,^78^, ^79^, ^80^, ^81^, ^82^ Na_3_V_2_(PO_4_)_3_F_3_,^83^, ^84^, ^85^ Na_2_Fe_0.5_Mn_0.5_PO_4_F,^79^ NaVPO_4_F,^77^, ^86^ and Na_3_V_2_O_2_ *_x_*(PO_4_)_2_ F_3−_ *_x_*.^87^, ^88^ Among the various cathode materials, NASICON‐type materials have attracted broad attention. As a typical representative, Na_3_V_2_(PO_4_)_3_ (NVP) has been widely studied due to its attractive Na^+^ storage properties. Na_3_V_2_(PO_4_)_3_F_3_ (NVPF) is another promising NASICON‐type material that exhibits superior performance. The crystal structure of NVPF can be described as having a space group of *P*4_2_/*mnm* (crystallizes in tetragonal symmetry), with [V_2_O_8_F_3_] bioctahedron units bridged by [PO_4_] tetrahedral units, both of which contribute to the extended 3D framework. Hence, along the (110) and (001) directions, Na^+^ can migrate freely through the large tunnels (**Figure** **11**a).^83^, ^85^ Jiang et al. first reported the outstanding electrochemical performance of NVPF in Li‐ion batteries, which exhibited a reversible capacity of 127 mAh g^−1^ between 3.0 V and 4.5 V (vs. Li/Li^+^).^89^ Then, in Na‐ion batteries, it also delivered a specific capacity of 115 mAh g^−1^, with three different discharge plateaus at 3.3, 3.6, and 4.0 V (vs. Na/Na^+^).^90^ To further determine the phase‐reaction mechanism of NVPF, computations were combined with experimental studies. The results demonstrated that reversible sodiation/desodiation occurred via a one‐phase reaction and that the structure of NVPF remained quite stable upon extraction and insertion of sodium,^91^ which is shown by the ex situ XRD results in Figure 11b and c. In further research, the ion migration mechanisms were proposed via first‐principles calculations.^84^ It was demonstrated that two of the three Na sites, namely, the Na(1) and Na(2) sites, could be extracted and inserted into the structure via a two‐step electrochemical process. The cyclic voltammetry (CV) curve in Figure 11d further proves the oxidation/reduction and the process of phase transformation. The first two pairs of redox peaks around 3.3 V and 3.7 V reasonably account for the extraction/insertion from Na(2) sites, corresponding to a two‐step process, whereas the higher potential at approximately 4.2 V is due to the extraction of the second Na^+^ from the Na(1) sites. A capacity of nearly 130 mAh g^−1^ was obtained after 50 cycles between 4.3 and 2.0 V, and a long‐term cycling study showed that even after 1000 and 3000 cycles at 10 C and 30 C, respectively; capacity retentions of 70% and 50%, respectively, remained.

**Figure 12:**
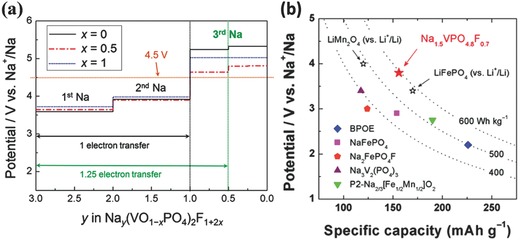
a)Voltage–composition curves for the Na_y_(VO_1−x_PO_4_)_2_F_1+2x_ (0 ≤ x ≤ 1; 0 ≤ y ≤ 3) electrodes from first‐principles calculations. Reproduced with permission.^93^ b) Energy density of Na_1.5_VPO_4.8_F_0.7_ compared with various cathode materials for NIBs and Li‐ion batteries. Reproduced with permission.^94^ Copyright 2013, American Chemical Society.

In addition to NVPF, a mixed‐valence family of compounds, Na_3_V_2_O_2_ *_x_*(PO_4_)_2_F_3−2_ *_x_*, between Na_3_V_2_(PO_4_)_3_F_3_ (*x* = 0) and Na_3_(VO)_2_(PO_4_)_2_F (*x* = 1) have also been proposed.^88^, ^92^ Here, *x* corresponds to the oxygen content in this material, which is closely related to the lattice parameter and cell volume of Na_3_V_2_O_2_ *_x_*(PO_4_)_2_F_3−2_ *_x_*. Ex situ XRD and X‐ray absorption near‐edge structure (XANES) analyses were used to illustrate that the electrochemical sodium extraction/insertion mechanism of Na_3_V_2_O_2_ *_x_*(PO_4_)_2_F_3−2_ *_x_* can be classified as a solid‐solution mechanism and that the vanadium oxidation state was +3.8. Furthermore, two factors contributed to the multi‐electron transfer: (1) different oxygen contents will result in the extraction of different Na ions below 4.5 V (vs. Na^+^/Na); and (2) the different redox potentials of V^3+^/V^4+^ and V^4+^/V^5+^ will cause disorder in the operating voltage, which may make the voltage step at *y* = 1.0 gentler.^93^ **Figure** **12**a shows the relationship between the potential and the composition of Na*_y_*(VO_1−_ *_x_*PO_4_)_2_F_1+2_ *_x_* (*x* = 0.0, 0.5, and 1.0) through first‐principles calculations. Keeping the plateau below 4.5 V and maintaining the intermediate phase at *y* = 1.0 is beneficial to multi‐electron transfer. It is worth mentioning that the novel cathode material of Na_1.5_VPO_4.8_F_0.7_ could exhibit a theoretical energy density of approximately 600 Wh kg^−1^ (3.8 V × 155.6 mAh g^−1^ = 591.3 Wh kg^−1^), corresponding to 1.2 electrons transferred per unit with the V^3.8+^/V^5+^ redox couple. Such a high energy density exceeds that of any other cathode material for Na‐ion batteries, as shown in Figure 12b. The electrochemical measurements show that it has a long‐term life, with 95% capacity retention for 100 cycles and ≈84% after 500 cycles.^94^

There is an ongoing quest for new, environmentally friendly iron‐based alkali fluorophosphates that can be operated on the Fe^2+^/Fe^3+^ couple and transported facilely for alkali ion‐like LiFePO_4_. A new material, Na_2_FePO_4_F, was first reported by Ellis and his group.^82^ It crystallizes in the *Pbcn* orthorhombic space group, and the bioctahedral [Fe_2_O_7_F_2_] units and [PO_4_] tetrahedral units share their corners to constitute a 2D framework. The two Na^+^ ions are located in both the [FePO_4_F] interlayers and near the sheets.^80^ To better understand how the atomic‐scale features influence the electrochemical properties of layered Na_2_FePO_4_F, a computational study using the atomistic simulation method was used to explore the Na‐ion conduction behavior and intrinsic defect properties.^95^ It was demonstrated that Na^+^ tended to migrate through the 2D network along the *a* and *c* planes due to the lowest energy pathways. The charge and discharge performance of the Na_2_FePO_4_F/C cathode material between potentials of 2.0 and 3.8 V (vs. Na/Na^+^) showed two reversible voltage plateaus, which are ascribed to two revers ible phase transformations: Na_2_Fe^II^PO_4_F ↔ Na_1.5_FePO_4_F ↔ NaFe^III^PO_4_F. The unit‐cell volume change from Na_2_Fe^II^PO_4_F to oxidized NaFe^III^PO_4_F is only 3.7%, and thus a lower strain de‐/intercalation process is expected. Through a soft template method followed by high‐energy ball milling, the nanostructured Na_2_FePO_4_F delivered a reversible capacity of 116 mAh g^−1^ (close to the theoretical capacity 124 mAh g^−1^) at 0.1 C. Even at 1 C after 200 cycles, approximately 80% of its initial discharge capacity and approximately 99.4% coulombic efficiency were observed.^78^

In addition to Na_2_FePO_4_F, other transition metal fluorophosphates phases have also been recently explored as cathode materials, such as Na_2_CoPO_4_F and Na_2_MnPO_4_F,^80^, ^96^, ^97^ both of which can be prepared by sol–gel, hydrothermal, and high‐temperature solid‐state routes. Na_2_CoPO_4_F showed a high voltage plateau near 4.3 V (vs. Na/Na^+^) and a discharge capacity of 107 mAh g^−1^ (with a theoretical capacity of 122 mAh g^−1^ based on 1 electron transfer per unit), with a theoretical energy density of 524 Wh kg^−1^.^97^

Na_2_CoPO_4_F is regarded as a good candidate for high‐voltage Na‐ion batteries. Na_2_MnPO_4_F has also been explored as a high voltage energy density cathode material. It exhibited initial discharge capacities of 140 and 178 mAh g^−1^ at 30 °C and 55 °C, respectively.^19^ After 20 cycles at 55 °C, it also delivered a reversible capacity of 135 mAh g^−1^. Unfortunately, the practical room‐temperature electrochemical performance of Na_2_MnPO_4_F has not been reported. Therefore, further investigation of Na_2_MnPO_4_F is still needed.

#### 3.3.2 Fluorosulfates

LiFeSO_4_F shows a higher voltage (3.6 V vs. Li) than LiFePO_4_ and can reduce the dependence on material optimization such as carbon coating and nanosizing.^98^ It thus has triggered interest in the use of fluorosulfates for Na‐ion batteries. However, due to the moisture sensitivity of sulfates, the preparation of such materials generally demands a non‐aqueous environment. The crystal structure of monoclinic NaMSO_4_F was first reported using both ionothermal and solid‐state syntheses at a low temperature of 300 °C for 9 h,^99^ which is contrary to the fabrication of triclinic‐based LiMSO_4_F phases.

Among the NaMSO_4_F compounds, only NaFeSO_4_F has been found to be electrochemically active (Fe^2+^/Fe^3+^) towards Li at 3.6 V. An atomistic modeling method was used to study the Na‐ion transport behavior, and the results indicated that NaFeSO_4_F is a 1D Na‐ion conductor with a relatively low activation energy of 0.6 eV.^100^ Nevertheless, LiFeSO_4_F is effectively a three‐dimensional (3D) lithium‐ion conductor. Therefore, the ionic migration in NaFeSO_4_F should be lower than that of LiFeSO_4_F.

To decrease the water solubility of sulfates, novel bihydrated fluorosulfates NaMSO_4_F_3_·2H_2_O (M = Fe, Co, Ni) have been proposed for the reported existence of NaMgSO_4_F_3_·2H_2_O, and it has been noted that the introduced dehydrate helps control the water solubility of the sulfates.^101^ However, the preparation of NaFeSO_4_F·2H_2_O under nitrogen has proven to be difficult because Fe^2+^ can be easily oxidized to Fe^3+^ in an oxygen atmosphere. In general, fluorosulfates are prepared through a two‐step method. First, optimized rapid heating of FeSO_4_·7H_2_O is used to produce the precursor FeSO_4_·H_2_O. NaF is then mixed in the structure, which substitutes F for O.^102^ By introducing benzene to form a benzene–water azeotrope, NaFeSO_4_F and NaFeSO_4_F·2H_2_O were prepared by a facile one‐step route, which dramatically shortened the reaction time and effectively avoided the tendency of Fe^2+^ to oxidize to Fe^3+^.^103^ Although no information on the electrochemical properties of these two materials have been published to date, such preliminary work could lay the foundation for further work on fluorosulfates.

### 3.4 Transition Metal Sulfates Na*_x_*M*_y_*(SO_4_)*_z_* (M = Fe, Mn, Co, Ni)

**Figure 13:**
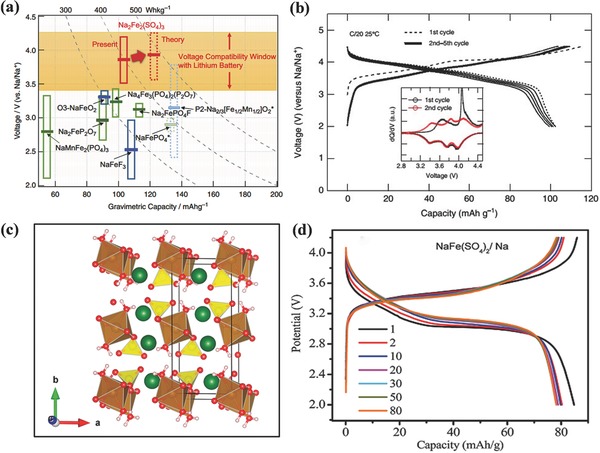
a) Overall comparison of the Fe‐based cathode materials that can function as Na sources in Na‐ion battery systems. b) Electrode properties of Na_2‐x_Fe_2_(SO_4_)_3_ in an Na cell. Reproduced with permission.^104^ Copyright 2014, Nature Publishing Group. c) Structural illustration highlighting the convoluted Na diffusion channels along the b‐axis; FeO_6_ octahedra are brown, SO_4_ tetrahedra are yellow, O atoms are red, and Na atoms are green. Reproduced with permission.^106^ Copyright 2014, American Chemical Society. d) Galvanostatic charge–discharge profiles at 0.05 C, subsequent cycles at 0.2 C rate for NaFe(SO_4_)_2_ vs. Na electrode. Reproduced with permission.^108^ Copyright 2015, Royal Society of Chemistry.

As compared to other polyanionic materials, sulfates possess stronger electronegativity, which contributes to higher redox potentials. Because of the thermal decomposition of SO_4_^2−^ above 400 °C, a low‐temperature solid‐state method below 350 °C was used by Yamada and his group to prepare an entirely new alluaudite‐type sulfate framework, Na_2_Fe_2_(SO_4_)_3_.^104^ Unlike typical NASICON‐related structures with corner‐sharing FeO_6_ octahedra, Na_2_Fe_2_(SO_4_)_3_ forms a unique alluaudite‐type framework, with edge‐sharing FeO_6_ octahedra. Then, the edge‐sharing FeO_6_ octahedra units bridge together by SO_4_ units, forming a 3D framework with large tunnels along the *c* axis. Thanks to the special structure, a very suitable operating potential of 3.8 V was observed based on the Fe^3+^/Fe^2+^ redox couple. To date, this is the highest potential among all Fe‐based Na‐ion battery cathode materials (**Figure** **13**a). A reversible capacity of 102 mAh g^−1^ (theoretical capacity 120 mAh g^−1^ based on 1 electron transfer) was obtained, even at a high of 20 C after 30 cycles, and a reversible capacity of 60 mAh g^−1^ was observed without any material optimization. The four different pairs of peaks in Figure 13b indicate the occurrence of some irreversible structural transformation, which is further demonstrated by the presence of off‐stoichiometry, such as Na_2+2_ *_x_*Fe_2−_ *_x_*(SO_4_)_3_.^105^ The sloping voltage curve then in turn indicates a single‐phase reaction.

Using a classical dissolution and precipitation route, Barpanda and his co‐workers successfully prepared another novel insertion compound, Na_2_Fe(SO_4_)_2_·2H_2_O.^106^ Figure 13c shows the crystal structure of Na_2_Fe(SO_4_)_2_·2H_2_O. Unlike the structure of bloedite, Na_2_Fe(SO_4_)_2_·4H_2_O, and anhydrous Na_2_Fe(SO_4_)_2_, the dihydrated Na_2_Fe(SO_4_)_2_·2H_2_O forms a pseudolayered monoclinic framework. Thanks to this unique structure, convoluted sodium channels develop and reversible ion intercalation occurs. To enhance the electronic conductivity, a 3D graphene‐based sandwich‐type Na_2_Fe(SO_4_)_2_·2H_2_O framework was constructed through a simple low‐temperature synthetic approach.^107^ It exhibited an insertion capacity of 72 and 69 mAh g^−1^ in sodium and lithium systems, respectively. Even at a high rate of 5 C, it delivered 81% and 70% of the capacity for Na‐ion and Li‐ion batteries, respectively.

Apart from the abovementioned sulfate polyanionic materials, other sulfate compounds such as NaFe(SO_4_)_2_ and Fe_2_(SO_4_)_3_ have also been reported as cathode materials for Na‐ion batteries.^108^, ^109^ As depicted in Figure 13d, the NaFe(SO_4_)_2_‐layered cathode material shows a reversible single‐phase reaction with the Fe^3+^/Fe^2+^ redox couple at a voltage of ≈3.2 V (vs. Na^+^/Na). After 80 cycles, a reversible discharge capacity of 78 mAh g^−1^ (theoretical capacity 99 mAh g^−1^ based on 1 electron transfer) was obtained at 0.1 C, with a coulombic efficiency of almost 100%.^108^ A rhombohedral NASICON compound, Fe_2_(SO_4_)_3_, has also been reported as a sodium intercalation host, where the tetrahedral‐corresponding SO_4_ groups share their corners with the octahedral‐corresponding FeO_6_ groups. The 3 V voltage plateau was ascribed to a single‐phase mechanism, and a first‐cycle discharge capacity of 65 mAh g^−1^ discharged to 2.0 V at 13 mA g^−1^. Contrary to lithium, only 1 mol of Na^+^ per unit mol of Fe_2_(SO_4_)_3_ can be electrochemically stored as compared to 2 mol.^109^ Even though the obtained practical electrochemical performance of NaFe(SO_4_)_2_ and Fe_2_(SO_4_)_3_ were not superior as compared to other cathode materials, they expand the research scope of intercalation chemistry and provide a new sub‐group of iron‐based polyanionic materials.

To study the impact of substitution on the structure of Na_2+2_ *_x_*Fe_2−_ *_x_*(SO_4_)_3_ and the electrochemical redox properties, it was demonstrated that Mn substitution in Na_2.5_(Fe_1−_ *_y_*Mn*_y_*)_1.75_(SO_4_)_3_ (*y* = 0,0.25,0.5, 0.75, 1.0) solid solutions could increase the voltage of the Fe^3+^/Fe^2+^ redox; however, it could also result in a capacity collapse due to Mn^2+^ inactivity.^110^ In an effort to enlarge the alluaudite family and further improve the redox reaction potential, a novel high‐voltage (4.4 V) cathode material, Na_2+2_ *_x_*Mn_2−_ *_x_*(SO_4_)_3_ (*x* = 0.22), was first reported by Dwibedi et al., who combined synergizing experiments with ab initio DFT calculations.^111^ Such a monoclinic framework belonging to the *C*2/*c* space group comprises Mn_2_O_10_ units sharing corners with SO_4_ tetrahedral units. To further determine the migration mechanism of Na atoms in the Na_2+2_ *_x_*Mn_2−_ *_x_*(SO_4_)_3_ structure, the activation energies were calculated extensively for four possible diffusion paths. It was revealed that the effect of Mn vacancies increased the activation energy of Na^+^ ions that hopped along the (001) channels in the Na_2+2_ *_x_*Mn_2−_ *_x_*(SO_4_)_3_ structure. However, this in turn leads to ionic diffusion, also in the (010) direction, and thus it is characterized as a 2D ionic diffusion mechanism.^112^

Some main issues remain for transition metal sulfates: (1) How can we balance low‐temperature synthesis methods with high purity or crystallinity? Thermal treatment temperatures below 400 °C may not be high enough to remove all the impurities, let alone allow for in‐situ carbon coating; (2) the relatively high operating potential requires a higher level of endurance; (3) a clear understanding of electrochemical processes is essential for further performance improvements for this important class of cathode materials.

### 3.5 Transition Metal Silicates Na_2_MSiO_4_ (M = Fe, Mn, Co)

Orthosilicates of transition metals with the general formula Na_2_MSiO_4_ (M = Fe, Mn, Co) have always attracted attention as possible cathode materials for Na‐ion batteries because of the advantages of abundant resources and non‐pollution, with the added possibility of exchanging two sodium atoms per formula, corresponding to theoretical capacities in excess of 278 mAh g^−1^. The first synthesis of Na_2_MnSiO_4_ was by a sol–gel method as an intermediate product to prepare the *Pn* space group of Li_2_MnSiO_4_ for Li‐ion batteries.^113^ However, no clear electrochemical performance of Na_2_MnSiO_4_ was reported by the authors of that study. Using an ionic liquid electrolyte, Hagiwara and his group successfully synthesized carbon‐coated Na_2_MnSiO_4_ by a sol–gel method. At a rate of C/10 (13.9 mA g^−1^) at 298–363 K,^114^ reversible capacities of 70, 94, and 125 mAh g^−1^ were obtained for a cell tested at 298, 323, and 363 K, respectively. The capacity of 125 mAh g^−1^ corresponds to 0.9 Na^+^ ions per unit formula that can be reversibly extracted and inserted from the Na_2_MnSiO_4_ crystal at 363 K. Nevertheless, the low phase purity and costly ionic liquid electrolyte may restrict practical application. In order to further investigate the Na^+^ ion migration mechanism in the Na_2_MnSiO_4_ structure, Na*_x_*Li_2−_ *_x_*MnSiO_4_ (*x* = 2, 1, 0) compounds were investigated through first‐principles calculations.^115^ Although the migratory ions changed from lithium to sodium, the calculated results indicated that the diffusion paths in Na_2_MnSiO_4_ were similar to those in Li_2_MnSiO_4_. It was noted that Na^+^ ion diffusion in the Na_2_MnSiO_4_ structure was even faster than Li^+^ ions diffusion in the Li analog.

In another work, Na_2_CoSiO_4_ was successfully prepared by a simple hydrothermal method.^116^ As the first material used as Na storage material, the Na_2_CoSiO_4_ electrode exhibited a specific capacitance of 249 F g^−1^ at current density of 1 A g^−1^ in a three‐electrode system and excellent cycle stability after 1500 cycles at a current density of 1 A g^−1^ in a Na‐ion capacitor. In an effort to further probe the feasibility of using transition metal silicates, density functional theory combined with X‐ray diffraction studies were recently used to investigate the zero‐strain crystal structures of sodium iron orthosilicates, Na*_x_*FeSiO_4_.^117^ The orthosilicates were characterized as a diamond‐like Fe–Si framework, which is considerably robust against Na insertion/extraction. A very slight volume change was also observed during cycling owing to the stable structure of the polymorphs. Just recently, the crystal structure of *F* $\bar{4}$3*m* Na_2_FeSiO_4_ via both the solid‐state method and the sol−gel method was reported for the first time.^118^ A reversible capacity of 106 mAh g^−1^ was obtained between 1.5 and 4.0 V at 30 °C. A potential plateau at 1.9 V corresponded to the Fe^2+^/Fe^3+^ redox reaction. When charging to 2.5, 3.5, and 4.0 V, the volume shrinkage of Na_2_FeSiO_4_ was only 0.5, 0.6, and 0.9%, respectively. These values are much smaller than that of the pristine material of other polyanion‐type materials: approximately 6.7% for olivine LiFePO_4_,^119^ and 4% for Na_4_Fe_3_(PO_4_)_2_P_2_O_7_.^74^ The superior structure stability contributed to the impressive cycling stability; with current densities of 10, 50, 100, and 200 mA g^−1^, capacity retentions of 95, 96, 91, and 94%, respectively, were obtained after 20 cycles.

The crystallization of phase‐pure Na_2_FeSiO_4_ is relatively troublesome as one has to avoid the presence of undesired sodium silicate phases such as Na_2_SiO_3_ or the partial oxidation of Fe^2+^ into Fe^3+^. In addition, the mechanism of sodium extraction/insertion in Na_2_FeSiO_4_ is rather complicated and still requires a significant amount of research. Moreover, the operating potentials of these materials are somewhat low, hence many strategies need to be researched in order to improve the potential, such as substituting a portion of Fe for Mn or Co. In any case, the low cost and zero‐strain property of Na_2_FeSiO_4_ makes the orthosilicates of transition metals such as Na_2_MSiO_4_ (M = Fe, Mn, Co) worth further research, which is definitely promising for large‐scale electrochemical energy storage (EES).

### 3.6 Other Polyanion‐Type Compounds

In addition to the abovementioned polyanion compounds, which have already been widely studied for Na‐ion batteries, other polyanion compounds such as carbonophosphates,^120^, ^121^, ^122^ amorphous polyanion compounds,^123^, ^124^, ^125^, ^126^ and molybdenates,^127^, ^128^ have also been researched.

**Figure 14:**
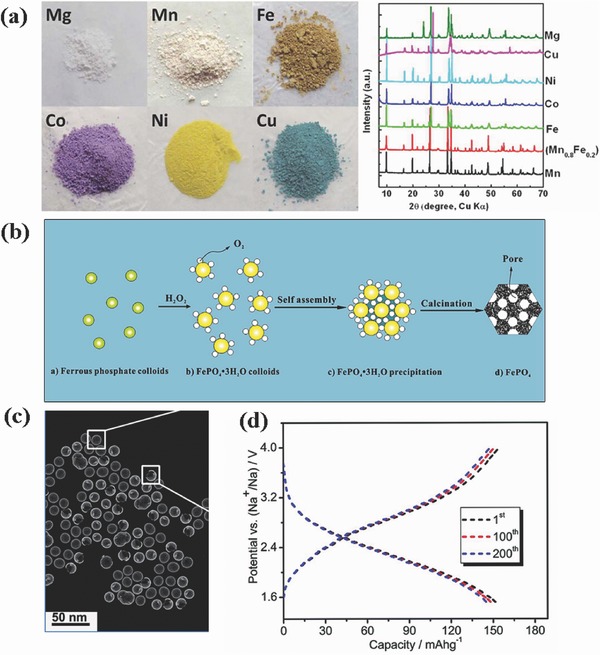
a) The colors of different Na_3_MPO_4_CO_3_ carbonophospahtes: M = Mg, Mn, Fe, Co, Ni, and Cu and XRD patterns of Na_3_MPO_4_CO_3_ carbonophosphates respectively. Reproduced with permission.^122^ Copyright 2012, American Chemical Society. b)An illustration of the formation mechanism of the mesoporous FePO4 nanospheres. Reproduced with permission.^124^ Copyright 2014, American Chemical Society. c) STEM image of the hollow amorphous NaFePO_4_ nanospheres. d) galvanostatic discharging–charging profiles of NaFePO_4_ nanospheres performed at a current density of 0.1 C. Reproduced with permission.^123^ Copyright 2015, Royal Society of Chemistry.

Carbonophosphates are a novel class of materials discovered through a high‐throughput ab initio computational approach. **Figure** **14**a shows six colors depending on different metal elements of the M site in the general formula Na_3_M(PO_4_)(CO_3_) (M = Mg, Mn, Fe, Co, Ni, Cu).^122^ The typical cathode material, Na_3_MnCO_3_PO_4_, has a high theoretical capacity of 192 mAh g^−1^ according to two‐electron transfer reactions. The charge and discharge profiles exhibit two plateaus that can be ascribed to the Mn^2+^/Mn^3+^ and Mn^3+^/Mn^4+^ redox reactions during Na^+^ intercalation/de‐intercalation, consistent with the prediction of the ab initio calculations.^128^ Prepared by a hydrothermal process and subsequent high‐energy ball milling with conductive carbon, the specific capacity of Na_3_MnCO_3_PO_4_ reached 176 mAh g^−1^ (92.5% of its theoretical capacity) by increasing the conductive carbon content to 60 vol%.^121^ Further study on Na_3_MnCO_3_PO_4_ cathodes showed a positive correlation between ionic conductivity and specific capacity.^121^ Furthermore, both structural defects and the average particle size have been proven to have a significant impact on specific capacity. Therefore, strategies in this direction such as micro–nanometer processing, doping, or carbon coating should be investigated to improve the electronic conductivity of the cathode materials. Note that too much carbon content will in turn affect the rate performance, so how to realize a uniform carbon layer at low temperatures requires investigation in the near future. We think that other transition metal‐based carbonophosphates may also exhibit good electrochemical activity for Na‐ion batteries.

Two vanadium‐mixed polyanion electrodes, Na_7_V_4_(P_2_O_7_)_4_PO_4_ 130 and Na_7_V_3_(P_2_O_7_)_4_ 131 have also attracted significant attention. These two composites are considered promising cathode candidates due to their high operating potentials: near 4.0 V vs. Na^+^/Na and superior high rate long cycling capability (even at a high rate of 20 C and 3 C after 800 cycles, approximately 94% and 91% of the capacity, respectively, was maintained).

Amorphous polyanion compounds are likely to improve the structural stability and electrochemical kinetic performance. As compared to crystalline materials,^132^ an isotropic structure with no lattice limitations will typical enhance Na‐ion diffusion by offering special diffusion paths that are not highly anisotropic.^133^ For example, crystalline iron phosphates have so far exhibited poor electrochemical performance because the lattice frameworks either can only provide undersized channels for Na‐ion diffusion or lack available sites for Na ions to reside. However, amorphous FePO_4_, which was synthesized through a simple chemically induced precipitation method (Figure 14b),^126^ exhibited a high discharging capacity of 151 mAh g^−1^ at 20 mAh g^−1^ and 94% capacity retention ratio after 160 cycles, revealing excellent electrochemical properties for Na‐ion storage. Recently, hollow amorphous NaFePO_4_ nanospheres were successfully fabricated by a simple in situ hard template method.^123^ Figure 14c shows the morphology of the as‐prepared NaFePO_4_ composites, which are large‐scale uniform hollow spherical particles with an average diameter of approximately 20 nm. The charging–discharging voltage profile (Figure 14d) of the hollow NaFePO_4_ nanosphere cathode at different cycles indicates that the electrochemical process was stable during the sodium de‐intercalation/intercalation reactions. This discharge curve shows a typical pseudo‐capacitance‐type behavior, as signaled by a sloping discharge curve without a potential plateau.^134^ However, further research needs to be conducted to investigate the relationship between sodium ion de‐intercalation/intercalation and pseudo‐capacitive behavior during the charge–discharge process for hollow amorphous NaFePO_4_ nanospheres.

An anti‐NASICON molybdenate, Fe_2_(MoO_4_)_3_, has also been identified as a cathode material for rechargeable batteries due to its environmental friendliness and abundant iron resources. It was first fabricated by the magnetron sputtering method as a positive electrode material for Na‐ion batteries, and it exhibited a reversible capacity of approximately 91 mAh g^−1^.^128^ In an attempt to understand the reaction mechanism by combining analysis of the structural evolution with electrochemical characterization, it was demonstrated that Na^+^ intercalation proceeds via two‐stage solid‐solution insertion into the monoclinic structure.^127^ Such a “rotational distortion mechanism,” as well as the symmetry‐mode analysis method, could also be applied to other polyanion‐type compounds.

## 4 Strategies to Enhance the Electrochemical Performance of Polyanion‐Type Compounds

Despite the advantages associated with polyanion‐type compounds, the low electron conductivity is still a common disadvantage for unmodified materials, which limits the dynamics of charge transport and further increases the polarization of electrochemical reactions. Therefore, convenient and efficient ways to increase electron conductivity are necessary. In addition, the relatively high operating potential of polyanion‐type compounds may result in electrolyte decomposition. Significant effort has been made to overcome this problem, such as surface conducting modification, particle‐size reduction, and so on.^32^ Here, we summarize some effective strategies for enhancing the electrochemical performance of polyanion‐type compounds on Na‐ion batteries.

### 4.1 Building Conductive Frameworks of Carbon Matrices

**Figure 15:**
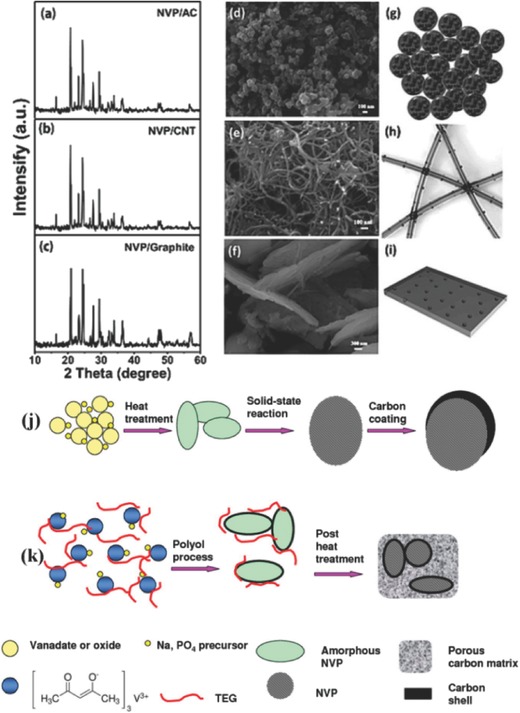
XRD patterns, SEM images, and schematic illustrations of Na_3_V_2_(PO_4_)_3_/AC (a,d,g), Na_3_V_2_(PO_4_)_3_/CNT (b,e,h) and Na_3_V_2_(PO_4_)_3_/graphite (c,f,i). Reproduced with permission.^31^ j) Conventional solid‐state process for microsized carbon‐coated Na_3_V_2_(PO_4_)_3_. k) Facile soft chemistry‐based double carbon‐embedding approach for (C@NVP)@pC. Reproduced with permission.^135^ Copyright 2014, American Chemical Society.

In an effort to improve the conductivity of polyanion‐type electrode materials, highly conductive carbon additives such as carbon black, graphite, or carbon fibers are often combined with active materials to form conductive frameworks of carbon matrices. Mai and his group dispersed Na_3_V_2_(PO_4_)_3_ nanograins in zero‐dimensional (0D), one‐dimensional (1D), and two‐dimensional (2D) carbon matrix nanostructures, which respectively correspond to acetylene carbon (AC) nanospheres (**Figure** **15**a), carbon nanotubes (CNTs) (Figure 15b), and graphite nanosheets (Figure 15c).^31^ It was demonstrated that Na_3_V_2_(PO_4_)_3_ dispersed in 0D AC nanospheres showed the best electrochemical performance of the three, whereas that of the CNT matrix was moderate and that of the graphite nanosheets was inferior.

In another work,^135^ a “double carbon‐embedding” Na_3_V_2_(PO_4_)_3_ framework was built by a facile in situ soft chemistry method with a post‐heat‐treatment procedure. The carbon‐coated nanosized Na_3_V_2_(PO_4_)_3_ particles were embedded in a highly effectively mixed conducting porous carbon matrix. Figure 15j and k respectively represent the conventional solid‐state method and the soft chemistry‐based method. The “double carbon‐embedded” Na_3_V_2_(PO_4_)_3_ showed ultrafast rate performance, which was comparable to that of a supercapacitor and to that of almost all lithium battery cathode materials. The performance can be ascribed to the highly conductive carbon matrix: (1) the porous carbon matrix functioned as an elastic buffer, mitigating the strain effects caused by volume changes during Na insertion and extraction; (2) the porous carbon matrix resulted in a 3D porous interconnected framework, facilitating electrical contact as well as Na‐ion conduction. The special method used to fabricate NaTi_2_(PO_4_)_3_ was also proven to be effective in fabricating Na‐ion battery anode materials.^50^

### 4.2 Element Substitution for Improving the Operating Potential and Na‐ion Diffusion Coefficients

As far as we know, large numbers of cations such as Mn^2+^,^136^ Y^3+^,^137^ Bi^3+^,^138^ Al^3+^,^139^ Mg^2+^,^140^ Fe^3+^,^141^ and Cr^3+^ have been successfully employed as dopants for Li_3_V_2_(PO_4_)_3_,^142^ and some positive impacts have been reported. However, with Na_3_V_2_(PO_4_)_3_, only Al^3+^,^36^ Mg^2+^,^35^ Cr^3+^,^33^ N,^39^ and B have been reported as dopants.^143^ It can be rationally deduced that other transition metal and non‐metallic elements should be studied to explore their functions on other polyanion‐type compounds beyond just Na_3_V_2_(PO_4_)_3._ It is worth noting that the doping of heterogeneous ions at either cation (Li^+^ and Fe^2+^) or anion (O^2−^) sites in LiFePO_4_ can greatly improve the electronic conductivity of materials in terms of capacity delivery, cycle life and rate capability.^144^, ^145^ Similarly, in the research for polyanion‐type cathode materials of Li_3_V_2_(PO_4_)_3_, different doping sites at Li^+^, V^3+^, and O^2−^ all proved to be effective.^139^, ^146^ However, research on non‐metal substitution of Na_3_V_2_(PO_4_)_3_ at P or O sites have rarely been reported except for small amounts on Na_3_V_2_(PO_4_)_3–_ *_x_*F_3_ *_x_*. More recently, B‐substituted Na_3_V_2_P_3−_ *_x_*B*_x_*O_12_ (0 ≤ *x* ≤ 1) as a stable cathode material for Na‐ion batteries has been presented. Combining experiments with DFT calculations, it was demonstrated that doping of B in P sites can change the local element valence, resulting in adjacent polyhedron geometry distortion, which narrows the band gap and facilitates the diffusion of Na^+^.^147^ Therefore, both metal and nonmetal substitution in different sites may have a significant impact on electrochemical performance. Additional studies in this direction should be carried out.

In an effort to prepare high‐potential cathode materials, introducing a higher redox couple into the pristine materials by element substitution has been proven to be an effective strategy for LiFePO_4_ cathode materials,^148^ which has also discussed in terms of sulfates on Na_2.5_(Fe_1−_ *_y_*Mn_I_)_1.75_(SO_4_)_3_.^110^ To probe the relationship between the electronic geometric structure and their electrochemical performance, different Fe/Mn ratios of alluaudite Na_2_Fe_3−_ *_x_*Mn*_x_*(PO_4_)_3_ microcompounds were obtained through solvothermal methods. The charge/discharge profiles demonstrated that the operating potential of Na_2_Fe_2_Mn(PO_4_)_3_ was approximately 0.5 V higher than that of Na_2_Fe_3_(PO_4_)_3_.^149^

### 4.3 Controlled Synthesis of Special Morphology to Optimize the Structure of Materials

We know that the morphology and crystal orientation of an electrode material will also significantly affect its electrochemical performance. Therefore, in order to retain fast ionic permeation and high electronic conductivity, as well as a stable structure for battery materials, new concepts of electrode structuring are still needed. In general, the controlled synthesis of nanoscale‐level materials has proven to be an effective strategy for increasing surface areas and shortening ion diffusion paths.

**Figure 16:**
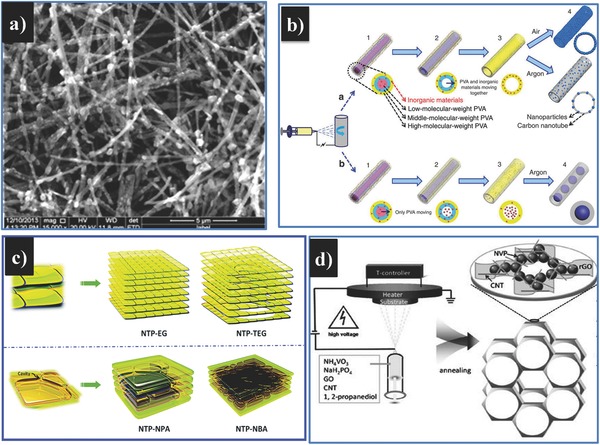
a) Apparent morphologies of Na_3_V_2_(PO_4_)_3_/C nanofibers. Reproduced with permission.^28^ Copyright 2015, Elsevier. b) Schematics of the gradient electrospinning and controlled pyrolysis method: preparation process of mesoporous nanotubes and pea‐like nanotubes. Reproduced with permission.^151^ Copyright 2015, Nature Publishing Group. c) Schematic illustration of the 3D nanoarchitectures of the four NTP products. Reproduced with permission.^152^ Copyright 2015, Royal Society of Chemistry. d) Schematic illustration of ESD technique to fabricate the interpenetrating 3D tricontinuous NVP: rGO‐CNT cathode. Reproduced with permission.^153^ Copyright 2016,Wiley‐VCH.

The electrospinning method is considered to be the most effective method to obtain nanofibers owing to the advantage of preparing continuous and uniform nanofiber materials.^150^ Recently, budding willow branch‐shaped Na_3_V_2_(PO_4_)_3_/C nanofibers were successfully synthesized by a simple electrospinning technique with poly(vinyl pyrrilidone) (PVP).^28^ This special morphology played a vital role in improving the cycle stability and rate capability of the electrode due to the conductive network built up by the nanofibers (**Figure** **16**a). In contrast, Mai and his group designed a gradient electrospinning and controlled pyrolysis method (Figure 16b) to synthesize various controllable 1D nanostructures and mesoporous nanotubes: pea‐like nanotubes and continuous nanowires.^151^ Owing to their large surface area, high conductivity, and robust structural stability, the prepared polyanion‐type compounds, Li_3_V_2_(PO_4_)_3_ and Na_3_V_2_(PO_4_)_3_, exhibited excellent electrochemical performance both in Li‐ion batteries and Na‐ion batteries.

Solvothermal and hydrothermal methods are also considered effective strategies to control the preparation of nanosized materials. Four NaTi_2_(PO_4_)_3_ nanocubes (Figure 16c) with controllable sizes were synthesized via a one‐pot solvothermal method with different organic precursors: ethylene glycol (NTP‐EG), n‐propanol (NTP‐NPA), n‐butyl alcohol (NTP‐NBA), and triethylene glycol (NTP‐TEG).^152^ The four as‐synthesized products showed high reversible capacities and excellent high‐rate performance as Na‐ion battery anodes.

Free‐standing materials have been of significant interest in recent years because conductive additives and binders observably lower the energy and power density. Based on a facile electrostatic spray deposition (ESD) technique, a self‐supported interpenetrating 3D tricontinuous cathode material (Figure 16d) Na_3_V_2_(PO_4_)_3_:rGO‐CN (reduced graphene oxide‐carbon nanotube), was prepared.^153^ The NVP:rGO‐CNT displayed outstanding rate capability and long cycling stability as both cathode and anode materials; even at a current density of 10 C, 96% of the initial capacity remained after 2000 cycles.

### 4.4 Designing and Selecting Suitable Electrolytes

Although polyanion‐type compounds have the advantage of high operating voltages, oxidation‐reduction reactions at high potentials are usually accompanied by electrolyte decomposition. As one of the most important part of batteries, the electrolyte is closely linked to the electrochemical performance and safety performance of batteries. Therefore, electrolyte optimization is a necessary and effective strategy to improving the electrochemical performance of polyanionic compounds. At present, organic, aqueous, ionic liquid, and polymer solid electrolytes are usually used for sodium ion batteries.

**Figure 17:**
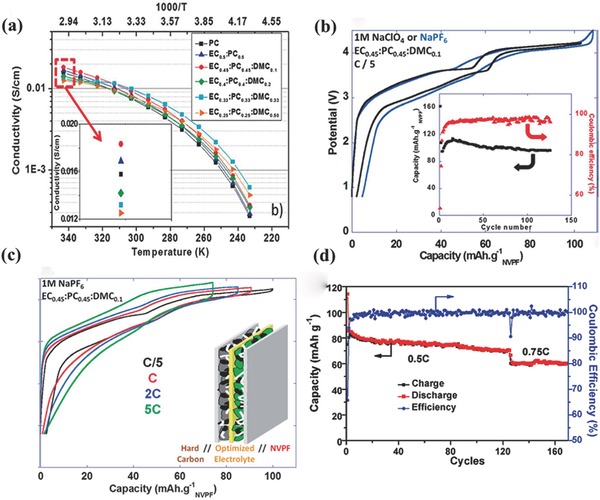
a) Arrhenius plots of the conductivity of the electrolyte based on 1 m NaTFSI salt dissolved in various solvent mixtures. b)Voltage versus capacity profiles for NVPF//HC full Na‐ion cells cycled in 1 m NaPF6 or 1 m NaClO_4_ in EC_0.45_:PC_0.45_:DMC_0.1_ recorded at C/5 (the inset displaysthe charge capacity and coulombic efficiency versus cycle number (C/5;1M NaClO_4_ in EC_0.45_:PC_0.45_:DMC_0.1_)). c) Voltage versus capacity profiles for NVPF//HC full Na‐ion batteries cycled in 1 m NaPF_6_ in EC_0.45_:PC_0.45_:DMC_0.1_ recorded at different rates. Reproduced with permission.^156^ Copyright 2013, Royal Society of Chemistry. d) the cycling performance of NaTi_2_(PO_4_)_3_/Na half cells with HMOP−PEO−NaTFSI solid electrolyte at 65 °C. Reproduced with permission.^159^

Jang et al. studied the effect of organic electrolyte systems (NaClO_4_/EC+PC and NaClO_4_/EC+DEC) on the electrochemical performance of Na_4_Fe_3_(PO_4_)_2_(P_2_O_7_).^154^ The results showed that the NaClO_4_/EC+PC electrolyte system delivered a relatively high reversible capacity of approximately 122 mAh g^−1^, even after 100 cycles. It also exhibited a high coulombic efficiency of 99%. However, the NaClO_4_/EC+DEC electrolyte system showed very low coulombic efficiency, with capacity loss during cycling after 60 cycles. This is because linear carbonates such as dimethyl carbonate (DMC), ethyl methyl carbonate (EMC), and diethyl carbonate (DEC) will severely decompose at the surface of Na metal electrodes and sodiated anodes. They will then diffuse to the cathode, resulting in electrolyte decomposition at high potentials of ≈4.2 V (vs. Na/Na^+^).^154^ However, the additive of fluoroethylene carbonate (FEC) allows the use of linear carbonates in Na‐ion batteries.^155^ In another work, Na_3_V_2_(PO_4_)_2_F_3_ was selected as the cathode material for electrochemical testing to evaluate its performance with an optimized electrolyte of 1 m NaClO_4_ dissolved in EC_0.45_:PC_0.45_:DMC_0.1_.^156^ The arrhenius plot in **Figure** **17**a shows that the most thermally stable composition with respect to DMC content is EC_0.45_:PC_0.45_:DMC_0.1_. Figure 17b and c show the voltage vs. capacity profiles of the full cell at different c‐rates, and the insets show the charge capacity and efficiency. An impressive rate performance and capacity retention are observed. It was also found that FEC is the only efficient electrolyte additive for Na cells among FEC, DFEC, VC, and ES, which are well known as film‐forming organic electrolyte additives in Li‐ion batteries. It was thus demonstrated that FEC additives can improve passivation and suppression of side reactions between Na metal and propylene carbonate solutions containing Na salts.^157^

**Figure 18:**
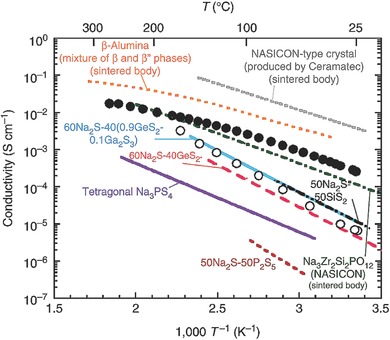
Conductivity of the Na_3_PS_4_ glass and glass‐ceramic electrolytes. Temperature dependences of the conductivities of the Na_3_PS_4_ glass (open circles) and the glass‐ceramic prepared at 270°C (solid circles). Conductivities of several Na^+^ ion conductors already reported are also shown as a comparison. Reproduced with permission.^158^ Copyright 2012, Nature Publishing Group.

All solid‐state batteries with inorganic solid electrolytes are promising power sources for a wide range of applications because of their safety and long cycle lifetimes. **Figure** **18** shows the electrical conductivities of Na_3_PS_4_ glass, glass–ceramic electrolytes, and typical inorganic solid electrolytes that have Na^+^ ion conductivities. The Na_3_PS_4_ superionic glass–ceramic electrolyte shows an ambient temperature conductivity of over 10^−4^ S cm^−1^. Although this conductivity seems to be one order of magnitude lower than that of sintered β‐alumina and the NASICON‐type crystal, the use of sulfide glass–ceramic results in good electrode–electrolyte contact by simple cold pressing. Furthermore, cyclic voltammetry measurements show that such a sulfide electrolyte has a wide electrochemical window of 5 V and is electrochemically stable against Na metal. Such room‐temperature operation of an all‐solid‐state (Na–Sn/Na_3_PS_4_/TiS_2_) cell represents the first step towards realizing practical all‐solid‐state Na‐ion batteries that are safe and inexpensive.^158^ Through a low cost and practical method,^159^ hollow mesoporous organic polymer (HMOP) spheres combined with oxide/poly(ethylene oxide) (PEO) can be pressed into a membrane to fabricate a solid electrolyte. Choosing the polyanion‐type LiFePO_4_ and NaTi_2_(PO_4_)_3_ as electrode materials to constitute all‐solid‐state cells, both of them show comparable electrochemical performance at an organic electrolyte and an average coulombic efficiency near 100%. (Figure 17d). In terms of safety, all‐solid‐state batteries are considered the most promising for future energy storage. How to improve the Na^+^ conductivity and reduce contact with electrode active materials are development directions toward realizing solid‐state Na‐ion batteries that operate at ambient or moderate temperatures.

## 5 Summary and Outlook

Na‐ion batteries are being considered one of the most suitable electrochemical power sources for large‐scale electrical energy storage due to the merits of abundant raw materials, significantly low cost, and relatively high specific capacity. The last five years have witnessed the rapid development of ambient‐temperature Na‐ion batteries. Among the different kinds of electrode materials for this system, polyanion‐type compounds such as phosphates, pyrophosphates, sulfates, silicates, carbonophosphates, and so on have attracted significant attention.

**Figure 19:**
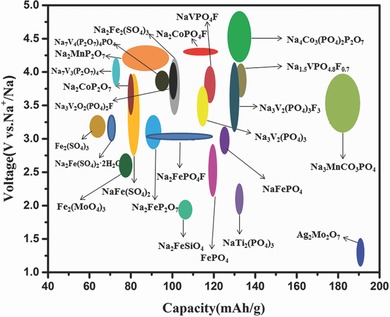
Recent acceleration in the number of reported polyanion compounds for Na‐based batteries. Voltage versus capacity for reported electrode materials in Na‐ion batteries, which have been drawn based on the data in Table 2.

In this review, we focused on the research progress of polyanion‐type compounds for Na‐ion batteries. By systematic analysis of the crystal structure, the electrochemical mechanisms, and problems to be solved, we can better optimize and design electrode materials for Na‐ion batteries, including new chemistry and new technology. The structure and electrochemical properties of polyanion compounds for Na‐ion batteries are listed in **Table** **2**. As can be seen, the potentials of these polyanion compounds (vs. Na/Na^+^) are distributed in a wide range from 1.4 V to 4.3 V, and their theoretical capacities range from 82 mAh g^−1^ to 278 mAh g^−1^. Therefore, in order to select a suitable polyanion compound for desired Na‐ion batteries, it is important to consider the working voltage and reversible capacity, as well as energy density, as shown in **Figure** **19**. It is notable that, although the practical capacities of polyanion compounds for Na‐ion batteries seem to be somewhat lower than those of their Li‐ion analogs, there is still room to span the gap between their actual and theoretical values. Na‐ion batteries have great advantages in terms of cost as compared to Li‐ion batteries, which will contribute to their application in large‐scale energy storage.

**Table 2:** Structure and electrochemical properties of current polyanion‐type electrode materials for Na‐ion batteries

Polyanion compounds		Structure	Potential/V (vs Na/Na^+^)	Theoretical/Practical capacity (mAh g^–1^)	Capacity retention
Phosphates	Na_3_V_2_(PO_4_)_3_ 32	NASICON‐type	3.4 V	117/113	20C, 70%(1000 cycles)
	NaFePO_4_ 15	olivine‐type	2.75 V	154.2/125	0.1C, 90%(240 cycles)
	NaFePO_4_ 16	maricite‐type	≈2.9 V	154.2/142	0.5C, 70%(200 cycles)
	NaMnPO_4_ 19	olivine‐type	≈3.75 V	155/85	0.05C, 55%(20 cycles)
	NaTi_2_(PO_4_)_3_ 46	NASICON‐type	2.1 V	133/131.2	10C, 89.3%(10000 cycles)
	FePO4^126^	amorphous	≈2.5 V	176/125	1C, 41.7%(300 cycles)
Pyrophosphates	Na_2_FeP_2_O_7_ 67	triclinic	3.0 V	97/91	1C, 74%(80 cycles)
	Na_2_MnP_2_O_7_ 68	triclinic	3.6 V	97.5/90	0.05C, 96%(30 cycles)
	Na_2_CoP_2_O_7_ 160	orthorhombic	3.95 V	96.11/80	0.1C, 86%(30 cycles)
	Na_7_V_3_(P_2_O_7_)_4_ 131	monoclinic	4.0 V	79.6/73	10C, 92%(100 cycles)
Mixed phosphates	Na_4_Co_3_(PO_4_)_2_P_2_O_7_ 73	orthorhombic	≈4.0 V	170/95	0.2C, 93%(50 cycles)
	Na_7_V_4_(P_2_O_7_)_4_PO_4_ 130	tetragonal	≈3.87 V	92.8/92.1	10C, 70%(1000 cycles)
Fluorophosphates	Na_2_FePO_4_F^95^	orthorhombic	≈3.0 V	124/116	1C, 80%(200 cycles)
	Na_3_V_2_(PO_4_)_3_F_3_ 85	tetragonal	3.75 V	128.2/130	10C, 70%(1000 cycles)
	Na_3_V_2_O_2_(PO_4_)_2_F^161^	sandwich	3.75 V	130/137.5	1C, 98.9%(40 cycles)
	Na_2_CoPO_4_F^97^	orthorhombic	4.3 V	122/107	61 mA g^–1^, 37.4%(20 cycles)
	Na_1.5_VPO_4.8_F_0.7_ 94	pseudolayered	3.8 V	129.7/134	0.1C, 95%(100 cycles)
Sulfates	Na_2_Fe_2_(SO_4_)_3_ 104	alluaudite	3.8 V	120/102	20C, 58.8%(30 cycles)
	NaFe(SO_4_)_2_ 108	layered	3.3 V	99/80	0.1C, 97.5%(80 cycles)
	Na_2_Fe(SO_4_)_2_ · 2H_2_O^107^	monoclinic	3.25 V	82/69	0.05C, 88.2%(20 cycles)
	Fe_2_(SO_4_)_3_ 110	rhombohedral	3.2 V	134/65	26 mA g^–1^, 20%(400 cycles)
Silicates	Na_2_MnSiO_4_ 114	–	≈2.8 V	278/125	–
	Na_2_FeSiO_4_ 118	cubic	1.9 V	276/106	200 mA g^–1^, 94%(20 cycles)
Carbonophosphates	Na_3_MnCO_3_PO_4_ 121	sidorenkite	≈3.7 V	192/176	–
Molybdenates	Fe_2_(MoO_4_)_3_ 128	monoclinic	≈2.6 V	Practice 79	–
	Ag_2_Mo_2_O_7_ 162	triclinic	1.4 V	Practice 190	20 mA g^–1^, 55%(1000 cycles)

Because layered transition metal oxides are usually accompanied by large volume changes during the Na insertion/extraction process, the characteristics of zero‐ or low‐strain polyanion‐type electrode materials are suitable as long‐life and high‐safety materials for practical Na‐ion batteries. To better cope with the intrinsic low electronic conductivity of polyanion compounds, some strategies have been used to enhance the electrochemical performance of such compounds: (1) building conductive frameworks of carbon matrices; (2) element substitution to improve the operating potential and Na‐ion diffusion coefficients; (3) controlled synthesis of a special morphology to optimize the structure of materials; and (4) designing and selecting suitable electrolytes. The future development of polyanion materials is expected to address some issues, such as (1) material genetic engineering combined with experimental science to select suitable materials for Na storage; (2) using material science and electrochemical technology to design and achieve special preferred orientation or morphology; (3) further improving the electrochemical reaction kinetics. Along with the urgent demand for large‐scale energy storage, the development of Na‐ion batteries is opening a new era full of opportunities and challenges, and the development of key materials is the main driving force. Better understanding and development of polyanion materials will result in strong support for promoting the commercialization of Na‐ion batteries.
